# Polarity-Selective Assembly Enables Tough and Stretchable Ionogels for Wearable Electronics

**DOI:** 10.1021/acsnano.6c02932

**Published:** 2026-06-23

**Authors:** Hongbo Fu, Xia Peng, Kaaviah Manoharan, Xiaohui Ju, Sanjay Kumar, Martin Pumera

**Affiliations:** † Future Energy and Innovation Laboratory, Central European Institute of Technology, 48274 Brno University of Technology, Purkyňova 123, Brno 612 00, Czech Republic; ‡ Advanced Nanorobots & Multiscale Robotics Laboratory, Faculty of Electrical Engineering and Computer Science, VSB - Technical University of Ostrava, 17. Listopadu 2172/15, Ostrava 70800, Czech Republic; § Department of Medical Research, China Medical University Hospital, China Medical University, No. 91 Hsueh-Shih Road, Taichung 40402, Taiwan

**Keywords:** solvent evaporation, polarity selectivity, phase separation, tough ionogel, ionic electronics

## Abstract

The widespread adoption of stretchable ionogels for emerging soft iontronics has been impeded by an inherent trade-off between mechanical robustness and ionic conductivity. Moreover, most existing approaches rely on intricate molecular designs or multistep processing, posing significant challenges to scalability and processability. Herein, we develop a facile and generalizable solvent-evaporation-induced phase-separation strategy that yields ionogels with superior toughness and ionic conductivity. By rationally pairing the ionophilic thermoplastic polyurethane (TPU) and ionophobic poly­(styrene-ethylene/butylene-styrene) (SEBS), a bicontinuous phase-separated architecture spontaneously emerges, guided by their polarity selectivity in ionic components and solvents. In this architecture, the ionic species preferentially partition into TPU-rich phases, establishing noncovalently confined ion transport networks, while ionophobic SEBS-rich phases impart mechanical reinforcement and structural stability without compromising ionic conductivity. The well-designed ionogels exhibit high stretchability (over 1500%), tensile strength (12.8 MPa), toughness (86.1 MJ m^–3^), and ionic conductivity (1.97 × 10^–2^ S m^–1^), as well as favorable elasticity and recyclability. Multimodal ionic skins fabricated with these tough and stretchable ionogels demonstrate sensitive and reliable responses to strain, pressure, and temperature stimuli, alongside the possibility to assemble wearable supercapacitors to power wireless gas-sensing devices. This fabrication strategy can be generalized to a variety of polymer-ionic systems, with potential applications in next-generation soft and wearable iontronic devices.

## Graphical abstract

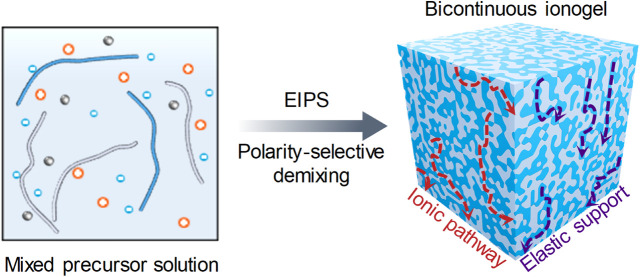

## Introduction

Stretchable ionogels have emerged as pivotal components in next-generation soft iontronics, including ionic skin,^1–4^ energy storage devices,^5–7^ and soft robotics,^8,9^ owing to their unique combination of nonvolatility, optical transparency, and robust physicochemical stability. However, conventional ionogels fabricated through swelling polymer networks with ionic liquid often suffer from fundamental limitations in simultaneously achieving satisfactory mechanical and ionic conductivity, typically exhibiting low fracture strength (<1 MPa), toughness (∼1,000 J m^–2^), elasticity (<100% strain), and ionic conductivity (<1 × 10^–3^ S m^–1^).^10,11^ These bottlenecks in performance primarily originate from the irreversible interactions between ionic components and the polymer matrix, resulting in an inherent conflict between ionic conductivity and mechanical resilience.^12^ Specifically, although increasing the content of ionic components enhances ionic conductivity, it simultaneously gives rise to plasticization that softens the polymer network and screening effects that disrupt dynamic bonding interactions, ultimately compromising mechanical integrity.^13–15^

Nature-inspired phase-separation strategies, governed by the interplay between entropic and enthalpic forces, offer a promising paradigm for achieving both excellent electrical and mechanical properties in ionogels.^16,17^ Recent advances have shown that polymerization-induced phase-separation strategies can yield ionogels that effectively decouple mechanical robustness and ion transport efficiency.^10,18–23^ Nevertheless, these approaches typically rely on intricate molecular designs and syntheses of functional polymers, which pose great challenges for synthetic scalability and material recyclability. Alternatively, ionogels can be fabricated through solvent processing methods such as solvent exchange or solvent-evaporation-induced phase-separation strategies. In solvent-exchange-induced phase-separation methods, multiple steps and prolonged processing times are typically required to fully replace a volatile solvent with a nonvolatile ionic liquid.^24^ By contrast, solvent-evaporation-induced phase-separation offers a more straightforward route to prepare tough ionogels. Moreover, because the polymer matrix remains soluble in appropriate solvents, most ionogels produced by this solvent-evaporation approach are recyclable. Although promising, the up-to-now trials for tough ionogels prepared via the solvent-evaporation-induced phase-separation strategy are unsatisfactory.^25,26^ The main reason is that the prevailing methods typically rely on simply blending ionic liquids with a single polymer matrix, and the ionic liquids act as strong plasticizers, showing a trade-off between the electrical and mechanical properties. Thus, it is highly desirable to develop a tough and recyclable ionogel via a solvent-evaporation-induced phase-separation process that can achieve comparable or even better performance than the ionogels fabricated by other strategies. Alternative recyclable polymers and breakthrough strategies in endowing the remarkable performance of ionogels are required to make the present soft iontronics more attractive and readily scalable to large-area manufacturing.

In this work, we report a facile and generalizable solvent-evaporation-induced phase-separation (EIPS) strategy that delivers ionogels with exceptional stretchability (over 1500%), remarkable tensile strength (12.8 MPa), superior toughness (86.1 MJ m^–3^), and high ionic conductivity (1.97 × 10^–2^ S m^–1^), along with exceptional elasticity and recyclability. The rationally designed tough and stretchable ionogel is composed of commercially available ionophilic thermoplastic polyurethane (TPU), ionophobic poly­(styrene-ethylene/butylene-styrene) (SEBS), and ionic components. The key to attaining these unique characteristics is the synergy of polarity-selective interactions between the polymer matrices and ionic components, coupled with the controlled kinetics of solvent evaporation, which drive the formation of the bicontinuous architecture. The ionic species are preferentially concentrated in the TPU-rich phases to establish noncovalently confined ion-transport pathways, whereas SEBS-rich phases resist plasticization by the ionic components and provide a robust mechanical framework, without sacrificing ionic conductivity in the resulting ionogels. Thus, this rational design can effectively decouple mechanical robustness from ionic conductivity, overcoming the long-standing trade-off in ionogels. To demonstrate the versatility of our tough and stretchable ionogel, we integrated it into multimodal ionic skins capable of sensing strain, pressure, and temperature, as well as into wearable supercapacitors that exhibit outstanding mechanical and electrochemical durability. Overall, the high-performance ionogels presented here provide a roadmap for the realization of tough and recyclable ionogels using a simple phase-separation strategy, which not only sheds light on the scalable and generalizable fabrication of advanced iontronic devices but also offer useful guidelines for developing recyclable ionogels with comparable or even better performance than ionogels made by more complex approaches.

## Results and Discussion

### Design and Implementation of Tough and Stretchable Ionogels

Figure 1a presents the compositional design and fabrication process of tough and stretchable ionogels with a bicontinuous structure, achieving a combination of ion transport and mechanical resilience. The emergence of the bicontinuous morphology was due to the ionic interaction and polarity contrast among the constituents. To clarify the mechanism, the chemical structures of the components for fabricating ionogels are summarized in Figure S1. Among them, TPU offers an ideal ion-conductive framework enabled by its intrinsic microphase separation between the soft and hard segments. The soft segment, composed of polyether chains, exhibits high segmental mobility and moderate polarity, which facilitate ion transport through dynamic ion-polymer interactions. By contrast, the hard segment domains, rich in urethane linkages, provide supramolecular reinforcement via hydrogen bonding and van der Waals interactions, contributing to mechanical robustness and maintaining the structural integrity of the ion-conductive network.^27^ Moreover, SEBS is a thermoplastic elastomer composed of polystyrene (PS) end groups and poly­(ethylene/butylene) (PEB) soft blocks, which provide excellent elasticity and elongation due to their covalently connected blocks, but their capacity for ion transport remains limited.^28,29^ In comparison, TPU provides mechanical durability and the ability to form efficient ion-transport pathways, whereas SEBS delivers excellent tensile strength and flexibility, making ionogels suitable for applications in wearable devices.^30^

**Figure 1:**
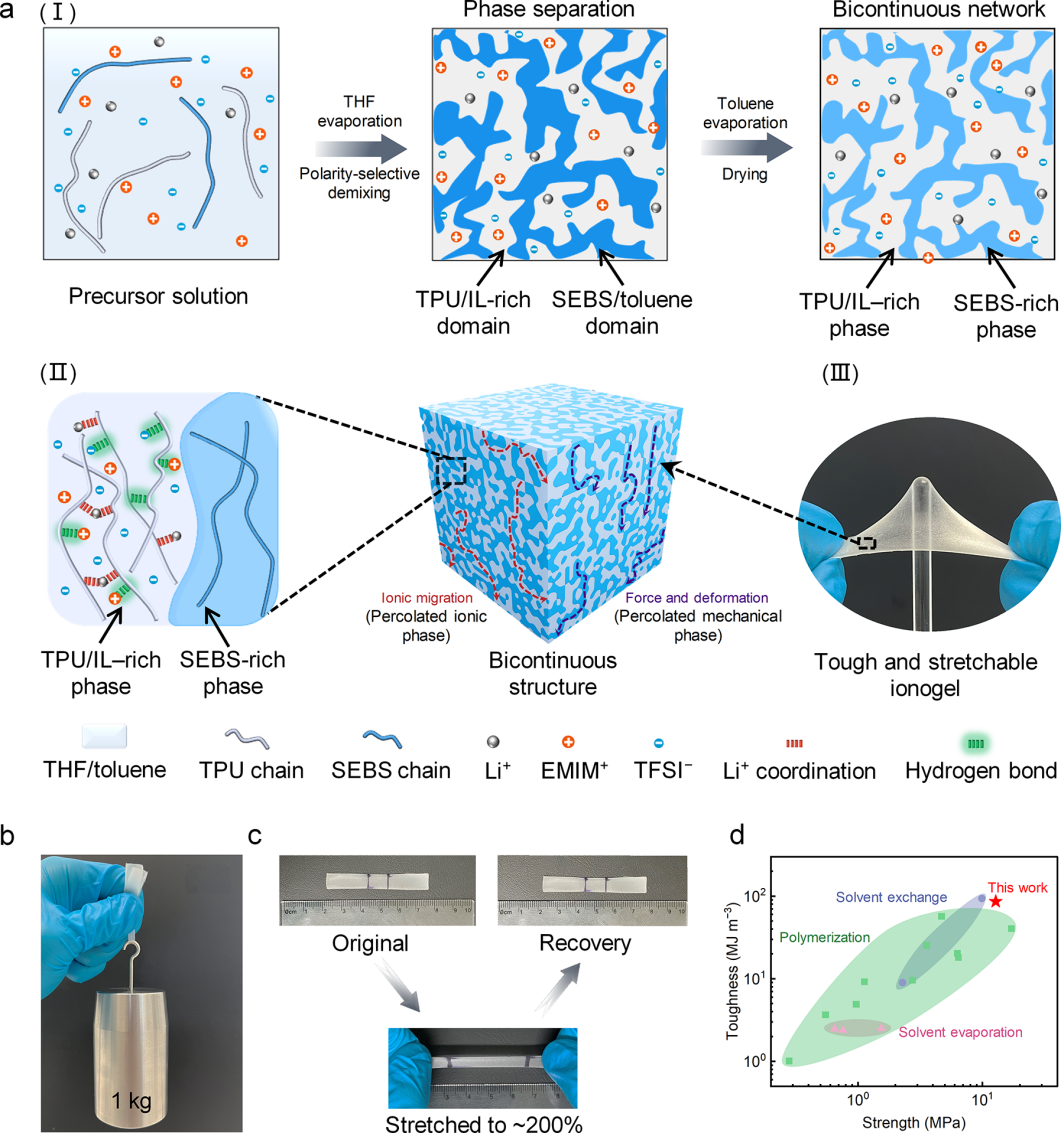
Design strategy and multiscale characteristics of phase-separated ionogels. (**a**) Schematic of the fabrication process and structural evolution of TSEL ionogels. (I) Illustration of a solvent-evaporation-induced phase-separation strategy. (II) Assembly driven by polarity-selective interactions and the resulting ion distribution. (III) Tough and stretchable TSEL ionogel with a bicontinuous structure. (**b**) Optical image showing that TSEL-40E/10L ionogel (cross-sectional area: 3.2 mm^2^) supports a 1 kg load without mechanical failure. (**c**) The stretchability of TSEL-40E/10L ionogels (thickness: 0.15 mm) and 10 min recovery from a strain of 200%. (**d**) The performance comparison of this work with reported high-performance ionogels prepared by polymerization,^18,20,23,34–40^ solvent exchange,^41,42^ and solvent evaporation^25,26,43^ in terms of toughness and tensile strength.

To preserve the structural and functional advantages of both polymers, we developed a simple and scalable EIPS strategy to construct a bicontinuous ionogel composed of TPU, SEBS, 1-ethyl-3-methylimidazolium bis­(trifluoromethylsulfonyl)­imide (EMIM TFSI), and lithium bis­(trifluoromethanesulfonyl)­imide (LiTFSI). A binary solvent system of tetrahydrofuran (THF, Snyder polarity index 4.0) and toluene (2.4) was used to dissolve the polymer components.^31^ This selective two-solvent strategy suppresses the macroscopic phase separation observed with THF alone, where the poor solubility of the PS blocks in SEBS causes a rapid demixing. Dissolving each polymer in its preferred solvent (TPU in polar THF and SEBS in nonpolar toluene) provides a balanced solvation environment. The miscible solvent pair, with closely matched solubility parameters, kinetically stabilizes the mixture and facilitates the formation of a uniform phase-separated morphology during casting. The addition of toluene also mitigates pore formation that would result from the rapid evaporation of THF and does not impede the development of the desired morphology owing to the incompatibility of toluene with the ionic constituents. Because of their distinct polarities, THF preferentially dissolves the polar TPU component, while toluene selectively dissolves the nonpolar SEBS.^32^ As the more volatile THF evaporates faster than toluene, a transient solvent gradient develops during drying, driving the mixture to segregate into a TPU/EMIM TFSI/LiTFSI (TPU/IL)-rich region and a SEBS-rich region (Figure 1a, **Domain I**). Coupled with strong and specific interactions between TPU and the ionic constituents, including Li^+^ coordination, ion-dipole interaction, and hydrogen bonding, drives the spontaneous formation of continuous TPU/IL networks. In parallel, the slower evaporation and diffusion of toluene allow SEBS chains to gradually self-assemble into a complementary elastic network (Figure 1a, **Domain II**). After complete solvent removal, these two percolating phases interpenetrate to form a well-defined bicontinuous network. In this final architecture, the TPU/IL phase provides efficient ion-transport pathways that enhance ionic conductivity, while the SEBS phase serves as a mechanical dissipater to maintain the integrity and elasticity of the TSEL ionogels by redistributing the stress and preventing crack propagation to the whole film during deformation^33^ (Figure 1a, **Domain III**). Thus, this phase-separation design simultaneously optimizes mechanical toughness and ionic conductivity in the TSEL ionogel.

To systematically investigate the morphology–property relationships, polymer matrices with various ratios of thermoplastic TPU and SEBS were fabricated via an EIPS strategy. A sample with a different TPU-to-SEBS mass ratio is termed TPU/SEBS = *a*:*b*, where *a* and *b* represent the weight fractions of TPU and SEBS, respectively. Subsequently, ionogels featuring distinct phase-separated microstructures were then prepared by introducing a controlled content of EMIM TFSI and LiTFSI into the selected polymer matrix. These samples are denoted as TSEL-*x*E/*y*L, where *x* and *y* represent the weight percentages of the EMIM TFSI and lithium salt, respectively. SEBS-free controls are labeled TEL-*x*E/*y*L, with *x* and *y* defined as described above. To demonstrate the generalizability of the EIPS strategy, ionogels fabricated by replacing SEBS with styrene-isoprene-styrene (SIS) block copolymer are defined as TIEL-*x*E/*y*L, with *x* and *y* defined identically. Ionogels prepared with 1-ethyl-3-methylimidazolium tetrafluoroborate (EMIM BF_4_) in place of EMIM TFSI are denoted TSEL-*x*B/*y*L, and the corresponding SEBS-free controls are TEL-*x*B/*y*L, where *x* and *y* correspond to the weight percentages of EMIM BF_4_ and LiTFSI, respectively.

As shown in Figure S2, the resultant TSEL ionogels exhibit high optical transparency across visible wavelengths, with transmittance ranging from 93% (TSEL-20E/10L) to 88% (TSEL-50E/10L), which arises from the favorable compatibility between EMIM TFSI, LiTFSI, and the TPU chain, coupled with the tailored phase-separated morphology that minimizes light scattering. The excellent mechanical robustness of these materials was shown by TSEL-40E/10L, which supported a 1 kg load that is around 4,000 times heavier than the ionogel itself without failure (Figure 1b). Furthermore, the TSEL-40E/10L ionogel (thickness: 0.15 mm) demonstrated good elastic recovery, returning fully to its initial state within 10 min after being subjected to a tensile strain of over 200% (Figure 1c). Moreover, Figure 1d and Table S1 compare the mechanical performance of TSEL-40E/10L with reported ionogels fabricated by polymerization,^18,20,23,34–40^ solvent exchange,^41,42^ and solvent evaporation^25,26,43^ for soft iontronics. The TSEL-40E/10L displays competitive toughness compared with previously reported ionogels prepared by either the same or an alternative strategy and attains a maximum (ultimate tensile) strength of 12.8 MPa that exceeds that of most ionogels produced by solvent-processing methods. Collectively, these mechanical properties position the recyclable TSEL ionogels fabricated via the straightforward EIPS strategy induced by polarity selectivity as a promising candidate for demanding applications in soft iontronics.

### Characterization of Polymer Matrix

To determine the optimal polymer matrix composition for ionogels, a series of TPU/SEBS blends with varied weight ratios was systematically evaluated. Given the difference in polarity between TPU and SEBS, the selective criteria focused on achieving a balance between morphological integrity and mechanical robustness for the preparation of ionogels.^22^ Fourier transform infrared (FTIR) spectra were performed to confirm potential molecular interactions between the TPU chain and the SEBS chain, as shown in Figure 2a. The characteristic absorption peaks corresponding to N–H stretching (3300–3400 cm^–1^), C=O stretching (1700–1730 cm^–1^), and styrene units (700–760 cm^–1^) exhibited negligible shifts with increasing SEBS content, which indicates the absence of strong specific interactions. In addition, differential scanning calorimetry (DSC) thermograms were collected for TPU/SEBS matrices with varying SEBS contents (Figure 2b), together with those of pure TPU and pure SEBS (Figure S3a). As the SEBS fraction increased, two distinct glass-transition temperatures (*T*_g_) at approximately −58 °C and −43 °C were simultaneously observed, which can be assigned to the soft segments in TPU and SEBS, respectively (Figure S3b). These results indicate the development of a phase-separated morphology in the TPU/SEBS blends. In detail, the thermodynamic immiscibility between polar TPU and nonpolar SEBS drives spontaneous phase separation with solvent evaporation, while the high molecular weights of both polymers kinetically restrict the interdiffusion. Additionally, the hydrogen bonds among TPU chains contribute to stabilizing the resulting morphology.

**Figure 2:**
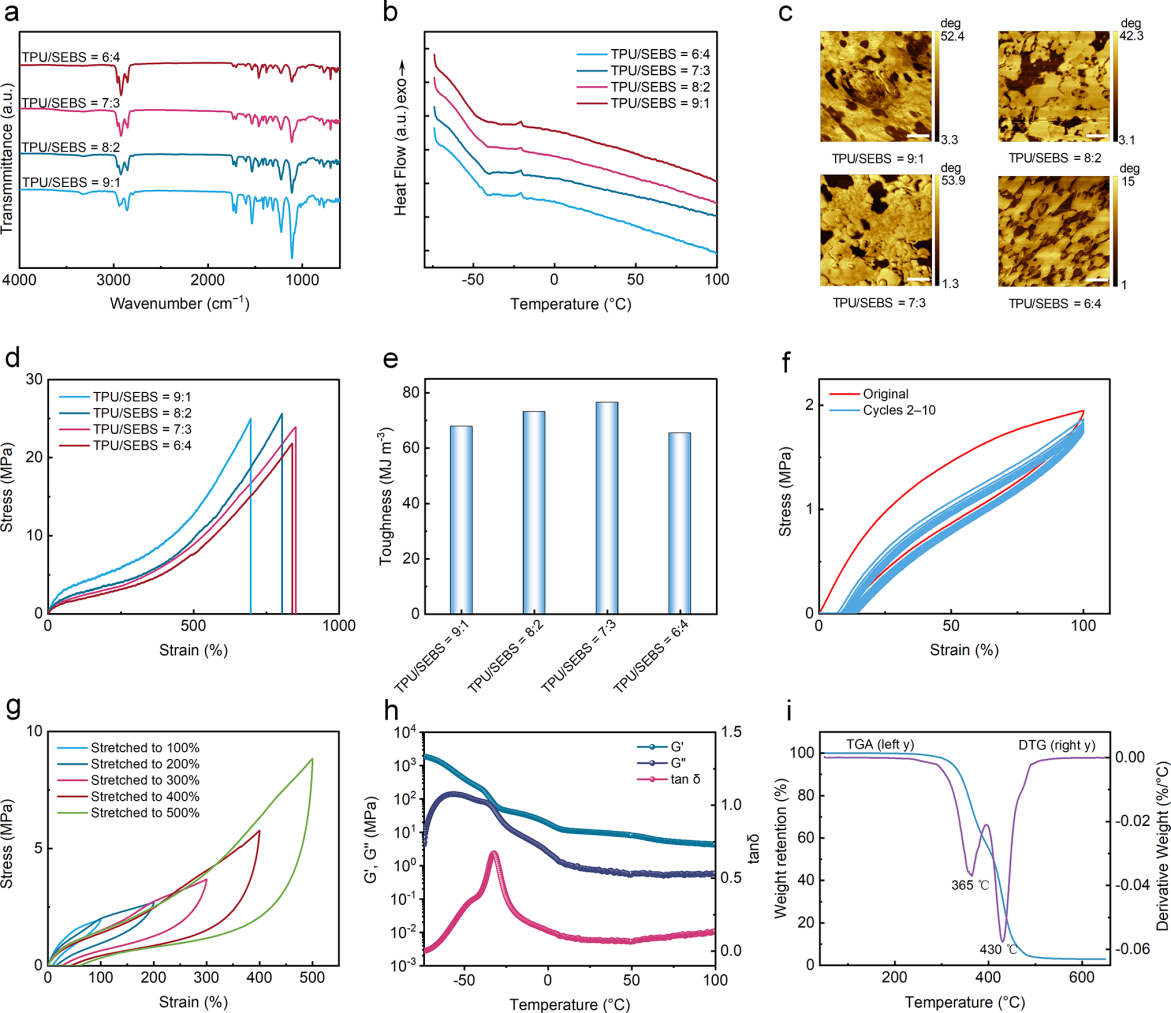
Morphology and mechanical properties of polymer matrices with varying SEBS content. (**a**) Fourier-transform infrared spectroscopy (FTIR) spectra of the polymer matrices. (**b**) Differential scanning calorimetry (DSC) traces of the polymer matrices. (**c**) Atomic force microscopy (AFM) phase images of the polymer matrices. The dark areas represent the SEBS-rich phase, and the bright areas represent the TPU-rich phase, indicating the phase-separated structure in the polymer matrices. Scale bar: 100 nm (**d**) Stress–strain curves of the polymer matrices. (**e**) Toughness of the polymer matrices. (**f**) Consecutive cyclic tensile curves of the TPU/SEBS = 7:3 matrix at a strain of 100%. (**g**) Successive cyclic tensile strain of the TPU/SEBS = 7:3 matrix at a strain ranging from 100% to 500%. (**h**) Dynamic mechanical analysis (DMA) temperature sweep curves of the TPU/SEBS = 7:3 matrix. (**i**) Thermogravimetric analysis (TGA) and derivative thermogravimetric (DTG) curves of the TPU/SEBS = 7:3 matrix.

Furthermore, the phase-separated structure in the systems was further confirmed by atomic force microscopy (AFM), as shown in Figure 2c. The AFM images reveal a well-defined distribution of the TPU phase (bright regions) and the SEBS phase (dark regions). Because of the polarity contrast between the TPU and SEBS, ionic interactions are predominantly localized within the TPU-rich phase. This phase-separated architecture preserves the distinct roles of each phase, providing an effective strategy to mitigate the inherent trade-off between mechanical robustness and electrical performance in ionogels. Notably, increasing the SEBS fraction progressively interrupts the continuity of the TPU-rich domains, which may compromise the percolation of ion-conducting networks and thereby impede the establishment of continuous ion-transport pathways in the resulting ionogels. The mechanical performance of the polymer matrices with different SEBS content was also investigated. As shown in Figure 2d, the tensile strength gradually decreases with increasing SEBS loading, which is attributed to the incorporation of the softer SEBS phase that disrupts the continuity of TPU-rich phases, thereby reducing their load-bearing contribution to the overall mechanical strength. Toughness reflects the capacity of materials for energy dissipation during deformation, which is correlated with both stretchability and mechanical strength. As calculated toughness shown in Figure 2e, the TPU/SEBS = 7:3 polymer matrix exhibits the highest toughness of 75.6 MJ m^–3^ among all tested samples. This enhanced toughness is attributed to the optimized phase-separated microstructure, enabling effective stress distribution and energy dissipation under mechanical loading.^44^ Among the tested results, the TPU/SEBS = 7:3 demonstrated an optimal combination of mechanical performance and a favorable phase-separated structure, making it the most suitable candidate for use as a polymer matrix of ionogels with phase-separated structures.

To further evaluate the mechanical performance of the TPU/SEBS = 7:3 matrix, 10 successive loading–unloading cycle tests at a strain of 100% (Figure 2f) and cyclic tensile tests under gradually increasing strains from 100% to 500% (Figure 2g) were performed, demonstrating the deformation and mechanical integrity. After a 30 min relaxation period, the loading–unloading curves at a 100% strain nearly overlapped with the initial cycle, indicating an almost complete recovery of both the hysteresis area and residual strain (Figure S4). Furthermore, dynamic mechanical analysis (DMA) measurement was conducted to measure the mechanical flexibility of TPU/SEBS = 7:3 under low and high temperatures (from −74 to 100 °C). Figure 2h shows that the value of the storage modulus (G′) remains higher than the loss modulus (G″) over the entire temperature range. By comparing the DMA responses of pure TPU and pure SEBS (Figure S5), the two distinct glass-transition temperatures (*T*_g_) observed in the blends can be assigned to the TPU-rich and SEBS-rich phases, respectively, further illustrating the phase-separated morphology. Moreover, the loss factor (tan δ) maximum at −32.4 °C indicates that the matrix maintains appreciable segmental mobility at subzero temperatures, highlighting its promising freeze-tolerant mechanical behavior for ionogel applications. It is worth noting that the *T*_g_ values obtained from DSC and DMA are not completely consistent. This discrepancy originates from the different physical quantities probed by the two techniques, with DSC being sensitive to changes in heat capacity and DMA reflecting the mechanical relaxation of polymer chains. Accordingly, the *T*_g_ determined from DMA is affected by the applied oscillation frequency, which introduces an additional observation time compared with that from DSC.^45^ The thermal performance of TPU/SEBS = 7:3 was analyzed by thermogravimetric analysis (TGA) and derivative thermogravimetric (DTG), as presented in Figure 2i. The sample remains thermally stable up to 300 °C, which ensures sufficient thermal tolerance for use as an ionogel matrix. The TGA/DTG curves exhibited two distinct degradation stages. Specifically, the first DTG peak (∼365 °C) is attributed to the hard segment in TPU, and the second peak (∼430 °C) corresponds to the SEBS backbone together with the soft segment in TPU, which is consistent with the phase-separated morphology described above. Overall, the TPU/SEBS = 7:3 matrix offers an optimal balance of properties and a well-defined phase-separated morphology, making it a robust platform for constructing high-performance phase-separated ionogels.

### Characterization and Toughening Mechanism of TSEL Ionogels

To elucidate the toughening mechanism of TSEL ionogels, we first investigated the influence of LiTFSI content. FTIR curves of ionogels with different ratios of LiTFSI (from 5 wt % to 20 wt %) were characterized (Figure S6a). The absorption peaks at 1703 cm^–1^ and 1732 cm^–1^ were attributed to the hydrogen-bonded and free C=O in TPU, respectively. With increasing the LiTFSI content, no obvious red shift of the C=O bands was observed. Instead, the peak at ∼1732 cm^–1^ weakened and eventually became unresolved, while the absorption around 1703 cm^–1^ became broader and more prominent (Figure S6b), which demonstrated the formation of Li^+^ ions coordinated with the carbonyl groups in the urethane hard segments.^46^ The ionic conductivity of the ionogels increased progressively with the LiTFSI content (Figure S7). Moreover, the stress–strain curves of the ionogels with various contents of LiTFSI, wherein mechanical strength and elongation at break increased from 5 wt % to 10 wt %, are attributed to the introduction of Li^+^-C=O coordination and hydrogen bonding (Figure S8). However, when the LiTFSI content reached up to 15 and 20 wt %, the mechanical strength of ionogels started to reduce, attributed to Li^+^-C=O coordination replacing stronger physical interactions in TPU chains, weakening the mechanical integrity while enhancing chain mobility and segmental flexibility at higher concentrations. Thus, to balance the mechanical and electrical performances in the following TSEL ionogel material systems, we selected the 10 wt % LiTFSI content for this study. Then, a series of TSEL ionogels was created by dissolving different amounts of EMIM TFSI (from 20 wt % to 50 wt %) and LiTFSI (10 wt %) into the TPU/SEBS = 7:3 polymer matrix and casting a film.

Scanning electron microscopy (SEM) images were applied to demonstrate the microstructure evolution of the TSEL ionogels, as presented in Figure 3a. All ionogels show a biphasic structure that gradually evolved with the increase in EMIM TFSI content from 20 wt % to 50 wt %. For TSEL-20E/10L, the morphology remained relatively homogeneous, indicating a miscible phase-separated system, which transferred into a distinct bicontinuous phase-separated structure in TSEL-30E/10L and TSEL-40E/10L. Moreover, the distinct phase domains became less when the EMIM TFSI content reaches 50 wt %, which is due to the plasticization effects caused by excessive content. It is evident that the composition of the TSEL ionogels includes characteristic features attributable to TPU and the anion from ionic additives as characterized by Raman spectroscopy (Figure S9). In addition, the SEBS-exclusive peak is hard to identify due to relevant polystyrene modes overlapping with aromatic vibrations from TPU and ionic components. Consequently, the Raman spectrum of the SEBS-free TEL-40E/10L (Figure S10a) shows only modest peak shifts relative to TSEL-40E/10L, rather than distinct SEBS-derived features. Furthermore, the gradual emergence and intensification of TFSI^–^ (∼740 cm^–1^) with the rising content of EMIM TFSI could be found. As shown in Figure 3b, a bicontinuous structure of the TSEL-40E/10L ionogel was confirmed by energy-dispersive X-ray (EDS) mapping, wherein the distinct sulfur (S) and oxygen (O) elements originated from the ionic components and TPU domains highlight isolated regions, and carbon (C)-rich areas devoid of oxygen correspond to the SEBS phase. By contrast, such a clear phase-separated architecture cannot be found in the EDS mapping of the SEBS-free TEL-40E/10L ionogel (Figure S10b). As shown in Figure 3c, AFM and Kelvin probe force microscopy (KPFM) were also employed to further corroborate the bicontinuous microstructure of TSEL-40E/10L ionogel. In conjunction with the SEM morphology and EDS mapping described above, the recessed domains are assigned to the TPU/IL-rich phase, whereas the raised regions correspond to the SEBS-rich phase. In the corresponding AFM phase image, brighter domains correspond to the TPU/IL-rich phase with a higher phase degree, and the darker domains are attributed to SEBS-rich phases, indicating that the SEBS phase has more elastic and effective stiffness. Thus, the SEBS-rich phase can serve as a mechanically robust scaffold that dissipates load and enhances the toughness of the ionogels. Notably, the phase contrast in the TSEL-40E/10L ionogel is significantly reduced compared to that of the pristine TPU/SEBS = 7:3 matrix (Figure 2c), consistent with ionic additives-induced plasticization of the TPU-rich phase and concomitant weakening of interchain associations. Furthermore, the observed domain sizes in the matrix and the ionogel exhibit significantly different values, which can be explained by the Flory–Huggins thermodynamics and phase-separation kinetics.^47,48^ Specifically, because of the preferential partition of ionic components into the TPU-rich phase driven by the stronger ionic interaction, the effective Flory–Huggins interaction parameter between the TPU-rich domain and SEBS-rich domain was enhanced, contributing to the thermodynamic driving force for larger phase separation. In addition, the incorporation of ionic components lowered the viscosity and raised diffusion, accelerating the coarsening kinetics of the phase-separated domains. Meanwhile, the KPFM potential map resolves phase-separated structures, attributed to the difference of local work function and interfacial dipoles arising from the accumulation of mobile ions (EMIM^+^/Li^+^ and TFSI^–^) and oriented dipoles in the polar TPU matrix, both localized in the TPU/IL-rich phase. Taken together, the coregistered surface topography, phase, and surface potential support a bicontinuous structure, in which the TPU/IL-rich phase provides the primary ion-conducting pathway, and the interpenetrating SEBS-rich phase affords mechanical reinforcement. This architecture likely arises from the limited compatibility between the ionic components and SEBS, coupled with the solvent-evaporation-induced self-assembly during film formation.^49^ This mechanism is supported by a comparison of the DSC traces of SEBS-free TEL ionogels (Figure S10c,d) with those of TSEL ionogels (Figure S11a,b). In detail, the distinct *T*_g_ (around −43 °C) of the SEBS phase is consistently observed in all TSEL ionogels, whereas it becomes indistinguishable in the TEL ionogels. These results indicate that the ionophobic SEBS-rich phase remains uncompromised even as the loading of ionic components in the complementary phase increases. Notably, upon the incorporation of SEBS, the phase-separated morphology introduces the larger interfacial area between the TPU/IL-rich and SEBS-rich domains in TSEL ionogels, giving rise to a more pronounced exothermic peak and further obscuring the *T*_g_ feature of the TPU/IL-rich phase in the TSEL ionogels. These observations demonstrate the simultaneous existence of the TPU/IL-rich phase and the SEBS-rich phase, as schematically illustrated in Figure 3d. To validate molecular interactions between the ionic additives and the polymer matrices, FTIR curves of TSEL ionogels with different contents of EMIM TFSI were recorded (Figure S12). Notably, the coordination interaction between Li^+^ ions and the carbonyl oxygen atoms in TPU still exists as described above. In addition, peaks associated with the TFSI^–^ anion, particularly the asymmetric and symmetric S=O stretching vibrations at around 1350 cm^–1^ and 1190 cm^–1^, exhibit slight shifts upon EMIM TFSI and LiTFSI incorporation, which may be due to the coordination between Li^+^ and the oxygen atoms in TFSI^–^, as well as their ion-dipole interactions with polar segments (like the N–H group in TPU) in the polymer matrix. Moreover, the peaks located at ∼3123 cm^–1^ and ∼3160 cm^–1^ are attributed to the C4,5–H and C2–H stretching vibrations of the imidazolium ring of EMIM TFSI, respectively, which indicates the hydrogen bond interaction between the imidazolium ring and the TPU matrix. These noncovalent ion-dipole interactions and hydrogen bonds among LiTFSI, EMIM TFSI, and TPU contribute to the formation of a phase-separated structure comprising the TPU/EMIM TFSI/LiTFSI phase. These interactions stably confine EMIM TFSI within the polymer networks, effectively preventing the leakage. Such structural confinement plays a pivotal role in regulating the ion transport behavior and mechanical integrity of the TSEL ionogels.

**Figure 3:**
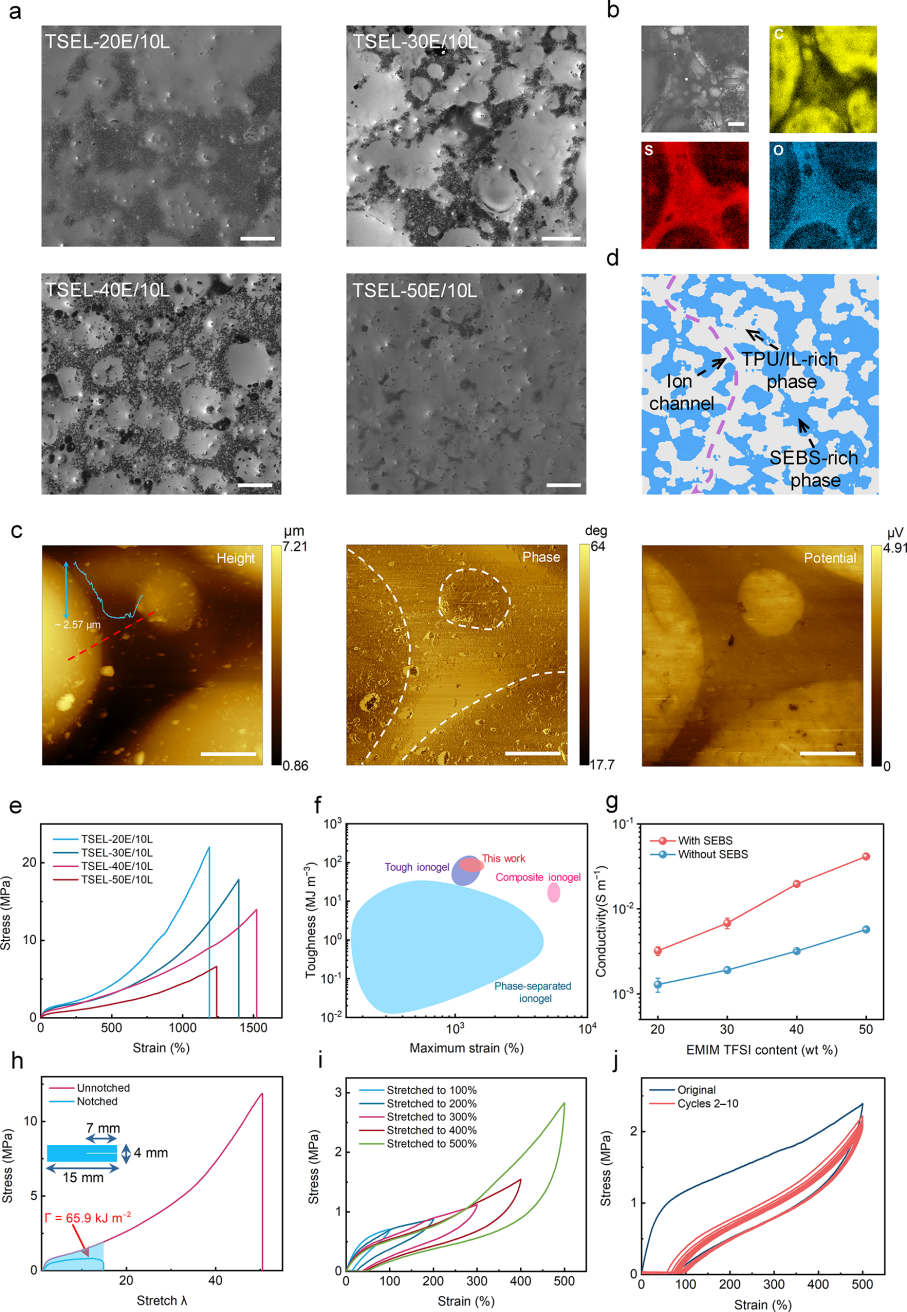
Morphology and characterization of various ionogels. (**a**) Section-view scanning electron microscopy (SEM) images of TSEL ionogels with varying EMIM TFSI content. Scale bar: 20 μm. (**b**) SEM image and corresponding energy-dispersive X-ray spectroscopy (EDS) elemental maps of carbon (C), sulfur (S), and oxygen (O) for the TSEL-40E/10L ionogel. Scale bar: 20 μm. (**c**) The AFM topography image with a representative line profile (blue) along the dashed trace, phase image, and Kelvin probe force microscopy (KPFM) potential map of TSEL-40E/10L ionogel corroborate the phase separation. Scale bar: 20 μm. (**d**) Schematic of the bicontinuous structure in TSEL ionogels. (**e**) Stress–strain curves of TSEL ionogels at different EMIM TFSI contents. (**f**) Mechanical performance of TSEL ionogels compared with previously reported ionogels in terms of toughness and stretchability. (**g**) Comparison of ionic conductivity between TSEL ionogels and SEBS-free TEL ionogels as a function of EMIM TFSI content. (**h**) Stress–strain curves of TSEL-40E/10L ionogels with and without notches. (**i**) Successive cyclic stress–strain curves of the TSEL-40E/10L ionogel at a strain range from 100% to 500%. (**j**) Consecutive cyclic tensile curves of the TSEL-40E/10L ionogel at a strain of 500%.

### Mechanical and Electrical Properties of TSEL Ionogels

Mechanical and electrical properties are crucial parameters for soft iontronics. Thus, the mechanical performance of the resultant TSEL ionogels was further evaluated by uniaxial tensile tests at room temperature. Figure 3e presents the tensile stress–strain curves of TSEL ionogels, suggesting that all TSEL ionogels exhibited exceptional stretchability and strength. Specifically, the TSEL-20E/10L ionogel shows a strain of 1189% and an ultimate strength of 22 MPa. Furthermore, when the EMIM TFSI content reached 40 wt %, the TSEL-40E/10L ionogel achieved a maximum strain of 1522% and an ultimate strength of 12.8 MPa. In comparison, the SEBS-free TEL-40E/10L ionogel achieved a strain of 896% and an ultimate strength of 16 MPa (Figure S10e). The increase of EMIM TFSI improves stretchability but reduces Young’s modulus, which can be attributed to a plasticization effect that interferes with polymer crystallization.^50^ This effect is supported by the X-ray diffraction (XRD) patterns (Figure S13). Specifically, a broad diffraction feature centered at 2θ ≈ 20° is observed across the series, which is assigned to the short-range of regular ordered structure of TPU-associated microphase-separated domains together with the contribution of the amorphous phase.^51^ Notably, the position of all peaks shows no appreciable shift upon incorporation of EMIM TFSI and LiTFSI, indicating that the average packing distance remains nearly unchanged. In contrast, the peak intensity decreases progressively, suggesting a reduced extent of ordered hard domains and an increase in the amorphous fraction.^52^ Figure S14 summarizes the calculated toughness of the TSEL ionogels. The toughness reaches a maximum of 89.4 MJ m^–3^ for TSEL-20E/10L and remains high at 86.1 MJ m^–3^ for TSEL-40E/10L but decreases upon further increasing the EMIM TFSI content. Significantly, the toughness of TSEL-40E/10L is 96.4% higher than that of the SEBS-free TEL-40E/10L ionogel (43.8 MJ m^–3^; Figure S10f). The toughness enhancement is attributed to the cooperative deformation of phase-separated domains and interfacial dissipation, which is led by the incorporation of the SEBS phase. This effect can be confirmed by comparing the DSC traces of the TSEL-40E/10L ionogel and the SEBS-free TEL-40E/10L ionogel (Figure S11c). Notably, an additional exothermic feature emerges in the TSEL-40E/10L ionogel between −67 °C and −58 °C, which is absent in the TEL-40E/10L ionogel. This feature suggests that the SEBS incorporation enhanced the phase separation and interfacial area, which together can provide additional pathways for energy dissipation. Figure 3f and Table S2 present a comparison of the mechanical performance of the TSEL-40E/10L ionogel with various ionogels, including phase-separated, tough, and composite types, for use in soft iontronics.^53–65^ The toughness of TSEL-40E/10L ionogel is prominent compared with reported phase-separated ionogels, while maintaining a maximum stretchability of 1522%, which is comparable to that of these systems. The J-shaped stress–strain curves of the ionogels resemble the mechanoresponse behavior of human skin.^66^ The calculated Young’s modulus of TSEL-40E/10L is approximately 3.31 MPa, which is well within the desirable range from 0.1 to 10 MPa for skin electronics, highlighting its potential for ionic skin (I-skin) applications.^67^ As shown in Figure 3g and Figure S15, the ionic conductivities of TSEL-20E/10L, TSEL-30E/10L, TSEL-40E/10L, and TSEL-50E/10L achieve 3.21 × 10^–3^ S m^–1^, 6.8 × 10^–3^ S m^–1^, 1.97 × 10^–2^ S m^–1^, and 4.13 × 10^–2^ S m^–1^, respectively. By contrast, the ionic conductivities of SEBS-free TEL-20E/10L, TEL-30E/10L, TEL-40E/10L, and TEL-50E/10L exhibit markedly lower conductivities of 1.29 × 10^–3^ S m^–1^, 1.90 × 10^–3^ S m^–1^, 3.1 × 10^–3^ S m^–1^, and 5.73 × 10^–3^ S m^–1^ under the same loading of ionic additives. Remarkably, the incorporation of the SEBS phase does not show a trade-off between electrical and mechanical properties, which benefited from the bicontinuous phase-separated structure. In the TSEL ionogels, the continuous TPU/IL-rich phase provides the effective ion-transport networks to enable their ionic conductivity, which also acts as a mechanical dissipater. Meanwhile, the highly stretchable ionophobic SEBS phase provides a percolated mechanical scaffold and spatial confinement of the conductive domains, thereby improving transport efficiency while maintaining the structural integrity of the ionogels. Moreover, a comparison of the ionic conductivities of the ionogels with and without LiTFSI reveals that the incorporation of 10 wt % LiTFSI significantly enhances the ionic conductivity across all TSEL ionogels (Figure S16). This improvement is attributed to the introduction of additional mobile charge carriers (Li^+^ ions), and the Li-bonding promotes the aggregation of EMIM TFSI in the TPU phase within the phase-separated structure, which serves as the effective ion transport pathway. Rational engineering of the bicontinuous architecture achieves a balance between mechanical robustness and ionic conductivity in the TSEL-40E/10L ionogel, which was selected for subsequent studies.

The fracture resistance of TSEL-40E/10L ionogel was further evaluated through pure shear tests (Figure 3h and Figure S17). The calculated fracture energy reached 65.9 kJ m^–2^, approximately 18 times that of the human stratum corneum (3.6 kJ m^–2^)^68^ and 6.6 times that of natural rubber (∼10 kJ m^–2^).^69^ The high tear resistance can be attributed to the phase-separated structure, wherein SEBS-rich phases mitigate stress concentrations and dissipate strain energy throughout the network, thereby preventing crack propagation. Moreover, elasticity is also an essential performance characteristic for ionogels, especially for the application of soft iontronics.^70^ Cyclic tensile tests were conducted on the TSEL-40E/10L ionogel to evaluate its elastic behavior under repeated loading–unloading cycles of constant amplitude. Figure 3i shows the single cyclic stress–strain curves of TSEL-40E/10L ionogel at gradually increased strain from 100% to 500%. The TSEL-40E/10L ionogel exhibits minimal hysteresis and residual strain under 100% and 200% tensile strain, indicating good elastic recovery. As the applied strain increases, both the hysteresis loop and residual strain become more pronounced, suggesting that the elevated strain energy at higher deformation levels leads to greater disruption of noncovalent interactions and the interface between the TPU/IL-rich and SEBS-rich phases (Figure S18). To further evaluate the elastic properties, 10 successive cycles at 500% strain were conducted, as shown in Figure 3j. The cyclic curves display a large hysteresis and a 90% residual strain. Specifically, the first cycle shows the larger hysteresis, and the hysteresis areas are significantly reduced in the following cycles, which is partially due to the unrecovered noncovalent interactions. Additionally, the cyclic stress–strain loops of TSEL-40E/10L ionogel show a reduced hysteresis area and residual strain compared with the SEBS-free TEL-40E/10L ionogel (Figure S10g), indicating that the incorporation of the SEBS phase effectively dissipates mechanical energy and improves cyclic stability. Furthermore, the loading–unloading cycles almost overlapped the first cycles after a 30 min relaxation (Figure S19), suggesting the excellent elasticity that can be attributed to the reversible deformation of the elastomeric SEBS phase, which can effectively help the stress dissipation and stabilize the softer TPU/IL-rich phase.^71,72^ Under tensile deformation, the SEBS-rich phase will be elongated to orient fibrillar features to dissipate energy, and the surface morphology becomes more homogeneous after release (Figure S20). In sum, the results indicate a phase-separation toughening mechanism for the TSEL ionogel. The reversible H-bonds and ion-dipole interactions in the TPU/IL-rich phase contribute to excellent energy dissipation capacity. Overall, the synergetic interplay between the TPU/IL-rich phase and SEBS phase, organized in a well-organized bicontinuous architecture, affords ionogels that combine excellent ionic conductivity with robust mechanical performance, indicating the potential application in soft iontronics.

### Environmental Stability and Recyclability

Environmental stability is an important indicator to explore the potential practical applications of ionogels. Benefiting from the nonvolatility and the inherent thermal resistance of ionic components, as well as the stabilized mechanical skeleton, the TSEL ionogels exhibited good thermal stability. As shown in Figure 4a, the decomposition temperatures (*T*_d_, temperature of 5% weight loss) in TGA curves of all TSEL ionogels exceed 252 °C. With the increase of EMIM TFSI content, the *T*_d_ of the TSEL ionogels gradually increases. Additionally, combining the TGA results of the polymer matrix (Figure 2i) and the SEBS-free TEL-40E/10L ionogel (Figure S10h), the slight weight loss in the initial stage is mainly due to the evaporation of ionic components, whereas the major weight loss at higher temperatures is attributed to the overlapping degradation of the polymer matrix and the ionic components.^73,74^ Furthermore, as presented in Figure 4b, the temperature-sweeping rheological results reveal that the storage modulus (G′) of TSEL-40E/10L ionogel remains consistently higher than the loss modulus (G″) over the wide temperature range from −74 to 100 °C, suggesting temperature-dependent changes without undergoing a gel–sol transition. The modulus variations are primarily governed by the thermal relaxation of polymer chains and reduced intermolecular interactions, indicating the absence of a phase transition across the investigated temperature range. It should be noted that the tan δ exhibits a single dominant peak in the TSEL-40E/10L ionogel. This behavior is attributed to the proximity of pure SEBS and SEBS-free TEL-40E/10L ionogel (Figure S10i and Figure S21), leading to strong overlap. Thus, TSEL-40E/10L ionogel shows a merged and broadened tan δ response, consistent with relaxation heterogeneity arising from interphase contributions and additional mechanical constraints imposed by SEBS domains. The tan δ reaches the peak at −22.03 °C, indicating that the mechanical flexibility can be well maintained above this temperature. As demonstrated in Figure 4c, the infrared thermal imaging results further confirm the robust thermal tolerance of the TSEL ionogel, which remains stretchable after being stored at −20 °C and 60 °C for 12 h. The temperature shift in samples is due to the change in the temperature of the environment. In addition, the mass stability tests are also shown in Figure 4d, in which the weight of TSEL-40E/10L ionogels remains unchanged after being placed at 25 °C in a vacuum oven and 100 °C in the oven without vacuum for 10 days, indicating the absence of EMIM TFSI liquid leakage, attributed to the ionic interaction between ionic components and TPU, as well as the physical confinement of the ionophobic SEBS phase. To assess long-term stability, the compositions of both TSEL-40E/10L and SEBS-free TEL-40E/10L ionogels were monitored by X-ray photoelectron spectroscopy (XPS) at day 0 and after 7 days. The survey spectra for all four conditions show the expected core-level peaks for C 1s (∼284 eV), O 1s (∼530 eV), N 1s (∼400 eV), S 2p (∼167–170 eV), and F 1s (∼688 eV) in Figure 4e. The corresponding atomic percentages for F 1s, N 1s, S 2p, C 1s, and O 1s at day 0 and day 7 are provided in Table S3. The atomic ratio of F 1s and S 2p is around 3, which matches the stoichiometry of the TFSI^–^ anion, confirming the assignment of these signals to TFSI^−^. A qualitative comparison of these spectra immediately reveals that the SEBS-free TEL-40E/10L ionogel exhibits much stronger F 1s and S 2p signals than the TSEL-40E/10L ionogel at both day 0 and day 7, indicating that the TSEL-40E/10L ionogel retains ionic liquid more effectively than the SEBS-free TEL-40E/10L. This improved retention is attributed to the physical confinement of the ionic liquid by ionophobic SEBS phases,^75^ as well as controlled solvent evaporation during preparation, which prolongs interactions between the TPU matrix and the ionic components. The ability of the TSEL-40E/10L ionogel to prevent ionic liquid leakage makes it more suitable for designing soft iontronic devices.

**Figure 4:**
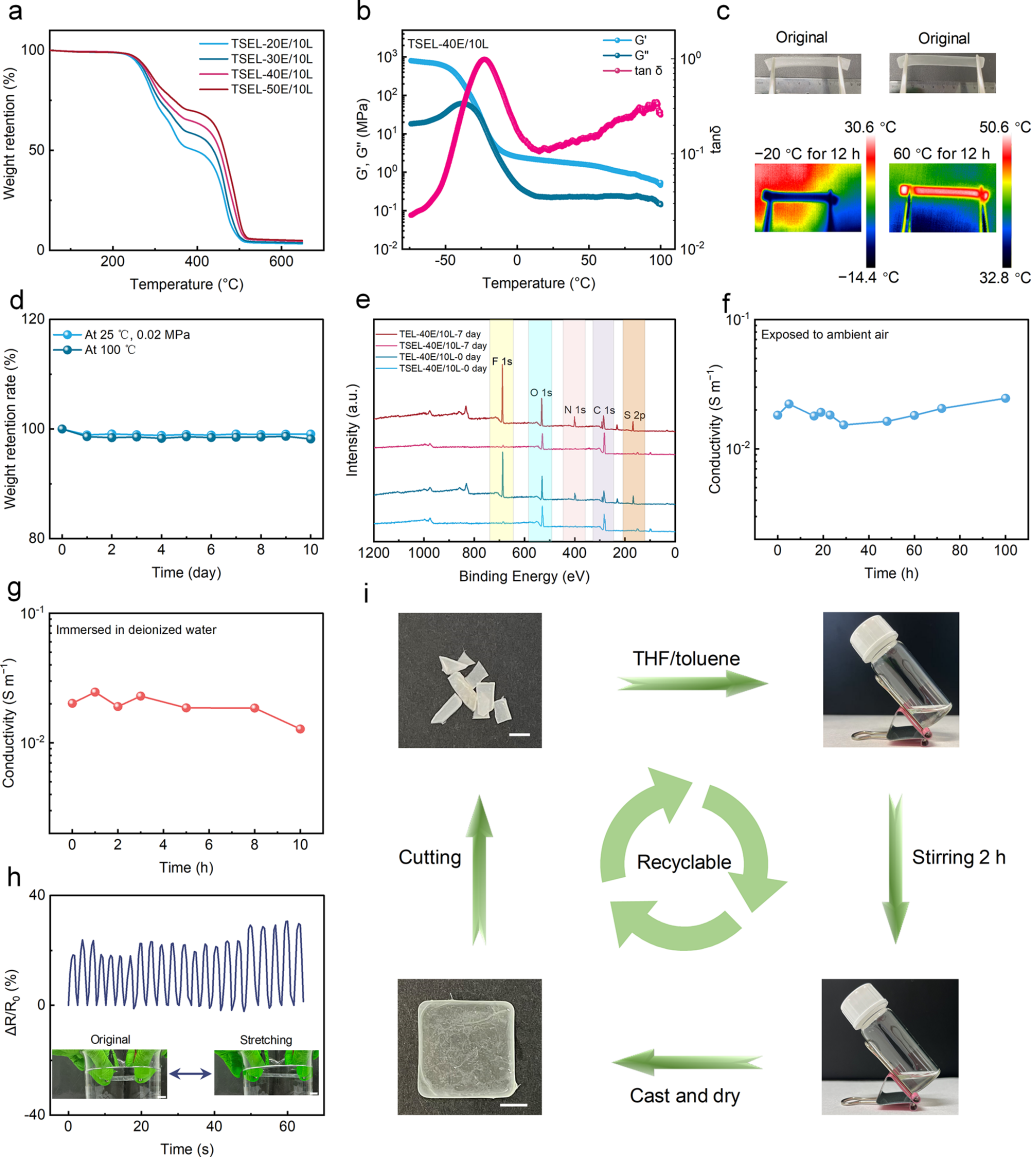
Environmental stability and recyclability of ionogels. (**a**) TGA curves of the TSEL ionogels. (**b**) DMA temperature sweep curves of TSEL-40E/10L ionogel. (**c**) Stretching the TSEL-40E/10L ionogel after storing at −20 and 60 °C for 12 h. Scale bar: 1 cm (**d**) Weight retention of TSEL-40E/10L ionogels stored at 25 and 100 °C for 10 days. (**e**) X-ray Photoelectron Spectroscopy (XPS) survey spectra of ionogels showing SEBS-assisted suppression of ionic liquid exposure. (**f**) Ionic conductivity of the TSEL-40E/10L ionogel as a function of air-exposure time. (**g**) Ionic conductivity of the TSEL-40E/10L ionogel as a function of water-immersion time. (**h**) Underwater strain-sensing performance of the I-skin. Inset: Photograph of the TSEL-40E/10L ionogel-based I-skin in the unstretched and stretched states. Scale bar: 1 cm. (**i**) Demonstration of the recyclability of the TSEL ionogel: cutting, dissolving in a THF/toluene solution, and reprocessing via solvent evaporation. Scale bar: 0.5 cm.

We further evaluated the environmental stability of ion transport and the electrical response of TSEL-40E/10L ionogel under diverse environmental conditions. As shown in Figure 4f, the ionogel exhibited long-term stability in open-air conditions for over 100 h. The slight fluctuations in ionic conductivity are likely attributable to moisture exchange with the ambient environment. To assess ion-transport performance over a broad temperature range, the ionic conductivities were measured from −20 to 70 °C. The ionogel maintained an ionic conductivity higher than 2.24 × 10^–3^ S m^–1^ across the entire tested temperature range (Figure S22a,b), indicating that efficient ion transport was retained even at low temperatures. The cyclic stability and recovery of ion transport were assessed by subjecting the ionogel to 15 heating–cooling cycles between 30 and 70 °C (Figure S22c). The conductivity–temperature characteristics exhibited good stability over continuous cycles, demonstrating the stable performance recovery under temperature cycling conditions. Furthermore, to investigate the electrical response under combined temperature and humidity variation, the relative resistance change (Δ*R*/*R*_0_) was monitored, where Δ*R* and *R*_0_ were the resistance change and the initial resistance, respectively. As shown in Figure S22d, the ionogel exhibited stable and repeatable resistance during cyclic humidity exposure at different temperatures. The decrease in resistance is due to moisture absorption by the ionogel, where the water molecules interact with the ionic species via ion-dipole interactions, thereby improving both ion solvation and ion mobility. Notably, the Δ*R*/*R*_0_ values were slightly lower at elevated temperatures than under room-temperature conditions, attributed to the reduced moisture absorption and accelerated water desorption from the ionogel.

Additionally, even when immersed in deionized water, the ionogel maintained a nearly constant ionic conductivity (Figure 4g) while exhibiting only a limited mass loss of approximately 10% over 8 h (Figure S23). On the basis of these characterizations, we explored potential scenarios of the TSEL ionogel as an underwater sensor. As shown in Figure 4h and Movie S1, stretch-release cycles underwater can be recorded continuously. Concretely, the resistance of the sensors decreased at the beginning of the experiment, which was due to the ionic rearrangement and enhanced ion mobility due to water-induced plasticization and solvation effects. Moreover, the reversible ionic-dipole interactions and hydrogen bonding in polymer chains contribute to the recyclable features of the TSEL ionogels. As shown in Figure 4i, the TSEL-40E/10L ionogel sample can be readily dissolved in the THF/toluene mixed solvent, recycled by the casting and drying processes, and then reprocessed into a designed specimen. To further evaluate the overall performance of the TSEL-40E/10L ionogel, representative previously reported TPU-based ionogels are summarized in Table S4. Previous TPU-based ionogels generally demonstrate an improved mechanical performance or specific environmental tolerance. In contrast, the TSEL-40E/10L ionogel exhibits superior integration of ionic conductivity, mechanical robustness, and environmental durability compared to most of these systems. These results highlight the advantage of the EIPS-induced phase-separated architecture, offering a practical material platform for recyclable, high-performance soft iontronic devices.

### Multimodal Sensing Performance of Ionogel-Based Ionic Skins

The TSEL-40E/10L ionogel demonstrates exceptional electrical properties, mechanical properties, and environmental stability, offering the possibility as I-skins. External stimuli such as mechanical deformation and temperature variations can dynamically alter ion redistribution and facilitate ionic diffusion within ionogels, enabling their multimodal sensing capabilities, including strain, pressure, and thermal responses. To evaluate the sensitivity of the ionogel-based strain sensor, the gauge factor (*GF* = (Δ*R*/*R*_0_)/ε, where ε denotes applied strain) was determined. As shown in Figure 5a, the Δ*R*/*R*_0_–ε curve displays good linearity and a stable sensing range over 0–600% strain, featuring two distinct slopes of 0.42 (region a, strain of 0–300%) and 0.94 (region b, strain of 300–600%). The resulting sensing performance is comparable to that of previously reported ionogel-based strain sensors.^76–78^ Cyclic loading–unloading tests under different strain levels (50–300%) confirmed the precise and repeatable resistance response of the strain sensor, highlighting the reliability of dynamic mechanical deformation, as shown in Figure 5b. Furthermore, the strain sensor exhibits stable responses over 500 consecutive loading–unloading cycles at 100% strain, demonstrating the durability of the ionogel for potential ionic-skin applications (Figure 5c). This stability is attributed to the excellent mechanical resilience and microstructural integrity of the TSEL-40E/10L ionogel. Building on the wide sensing range and excellent cyclic stability of the strain sensor, we next assessed its applicability in wearable motion monitoring. Benefiting from the mechanical robustness and skin-compatible modulus of the TSEL-40E/10L ionogel, the device was configured as an I-skin and conformally laminated onto human joints. As shown in Figure 5d, the I-skin attached to a finger produces reproducible resistance signals during extension and flexion over a range of actuation frequencies. Consistently, wrist and elbow motions were recorded reliably, as presented in Figure 5e and Figure 5f. When applied to the knee, the I-skin effectively tracked walking-induced joint motion through distinct resistance variations (Figure 5g).

**Figure 5:**
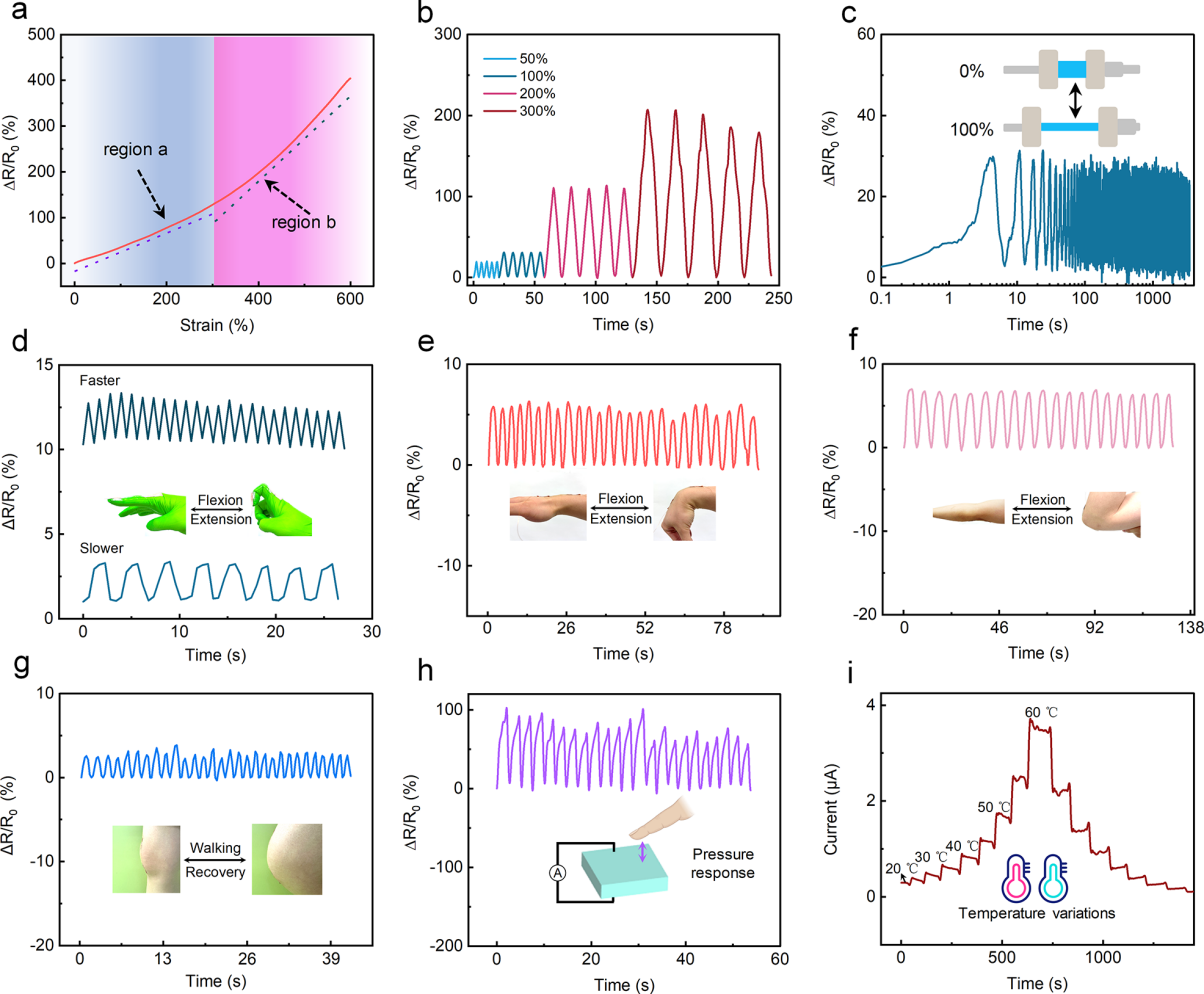
Multimodal sensing and electromechanical performance of the I-skins. (**a**) Relative resistance changes of the I-skin based on the TSEL ionogel as a function of strain. (**b**) Resistance changes under specific strains (50%, 100%, 200%, and 300%). (**c**) Durability test showing resistance changes over 500 successive loading–unloading cycles at 100% strain. (**d-g**) Strain-sensing response of the ionogel-based I-skin for human-motion monitoring: (**d**) finger, (**e**) elbow, (**f**) wrist, and (**g**) knee. (**h**) Resistance response of the ionogel-based I-skin under cyclic compressive loading–unloading. (**i**) Temperature-dependent current response of the ionogel-based I-skin under stepwise temperature variations.

Beyond tensile strain sensing, we further evaluated the ionogel I-skin for multimodal perception, including pressure and temperature sensing. Notably, the good resilience of TSEL-40E/10L ionogel enables fast recovery under cyclic compression with compressive strains from 10% to 60% (Figure S24). Accordingly, the I-skin exhibited a rapid and sustained response to manual compression, maintaining a stable signal output under the successive pressure stimuli (Figure 5h) as well as step loading (Figure S25). Furthermore, the same ionogel was integrated into a temperature sensor (Figure 5i), delivering stable and repeatable responses across a range from 20 °C to 60 °C with 5 °C increments. The observed increase in current with increased temperature is mainly attributed to the enhanced mobility of ions, which can be confirmed by the temperature-dependent increase in ionic conductivity (Figure S22a). Collectively, these results highlight the excellent multimodal sensing capability and mechanical adaptability of the ionogel-based I-skin.

### Wearable Supercapacitor Enabled by TSEL Ionogel Electrolytes

Beyond sensing, the combination of mechanical robustness and favorable electrochemical characteristics also renders the TSEL ionogel promising as a solid-state electrolyte for deformable energy-storage devices. Therefore, a flexible symmetric supercapacitor (FSC) was engineered through the rational integration of a polyimide (PI) film substrate, activated carbon (AC) cloth electrodes, and a freestanding TSEL-40E/10L ionogel membrane as the electrolyte, demonstrating multifunctional energy storage capabilities for wearable electronics.^79^ Figure 6a and Figure 6b illustrate cyclic voltammetry (CV) curves over different scan rates and the galvanostatic charge–discharge (GCD) profiles with different current densities for the fabricated flexible supercapacitor. The CV curves are symmetric at all scan rates and show the optimal capacitive behavior of the activated carbon with TSEL-40E/10L membrane. The maximum specific capacitance of 154 F g^–1^ is achieved at a current density of 0.5 A g^–1^, confirming the excellent ionic conductivity and efficient ion migration toward the electrolyte–electrode interface. Moreover, the fabricated flexible electrode exhibits a maximum specific capacitance retention of 96.8% even after 1,500 cycles, underscoring its outstanding electrochemical stability (Figure 6c). The superior performance is attributed to the optimized phase-separated architecture, in which the TPU/ionic components-rich phase provides complementary ion-transport pathways that promote efficient ion conduction, as discussed above.

**Figure 6:**
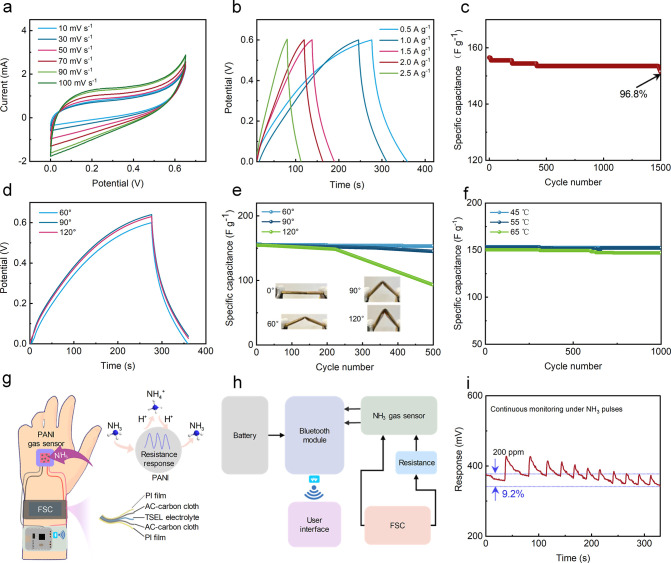
Electrochemical performance, mechanical reliability, and practical application of ionogel-based wearable supercapacitors. (**a**) Cyclic voltammetry (CV) curves of the flexible symmetric supercapacitor (FSC) at various scan rates. (**b**) Galvanostatic charge–discharge (GCD) curves at different current densities. (**c**) Cyclic stability of the FSC over 1500 cycles at 0.5 A g^–1^. (**d**) GCD profiles of the FSC under different bending angles at a current density of 0.5 A g^–1^. (**e**) Capacitance retention over repeated bending cycles at different angles. Insets: optical images of the FSC under bending angles of 60°, 90°, and 120°. (**f**) Cyclic stability test of the ionogel-based flexible supercapacitor at different temperature variations. (**g**) Schematic of a wearable supercapacitor-powered NH_3_ gas sensing system. (**h**) Block diagram of the integrated wireless system for powering and data transmission. (**i**) Potentiometric response/recovery transients of the NH_3_ gas sensor.

To evaluate the flexibility and potential application of the supercapacitor as a wearable power source, the flexible supercapacitor was systematically investigated through GCD analyses (Figure 6d) and CV curves (Figure S26) under different bending angles (60°, 90°, and 120°). All measured curves exhibited ideal capacitive behavior across various bending states, highlighting the excellent mechanical integrity and electrochemical reliability of the flexible supercapacitor, which benefited from the excellent mechanical properties of the TSEL ionogel-based electrolyte. Furthermore, angle-dependent capacitance retention was assessed over 500 charge–discharge cycles at the same bending conditions (Figure 6e), yielding capacitance retention rates of 98%, 94%, and 60% for 60°, 90°, and 120°, respectively. In addition to mechanical deformation tolerance, the thermal stability of the flexible supercapacitor was also evaluated, as shown in Figure 6f. The thermal-cycling test results over 1000 charge–discharge cycles unveiled capacitance retention of 92% and 88% at 45 and 55 °C, respectively, compared to 80% at 65 °C. The performance degradation at elevated temperatures is attributed to the synergistic effects of irreversible pore restructuring within the activated carbon electrodes and interfacial degradation caused by electrode drying. These results underscore the mechanical and thermal stability of the flexible supercapacitor, highlighting its strong potential for integration into real-world wearable electronic applications.

To demonstrate this potential in representative practical applicability, we developed a wearable wireless ammonia (NH_3_) gas-sensing device powered by the wearable supercapacitor. NH_3_ was selected as the target analyte due to its status as one of the most widely produced and utilized chemicals across various areas worldwide, posing notable environmental safety and human health concerns.^80^ In detail, the integrated platform consisted of the TSEL ionogel-based flexible supercapacitor, a polyaniline (PANI)-based NH_3_ gas sensor, an ESP32-C3 module with Bluetooth functionality, and a lithium polymer (Li-pol) battery, as schematically illustrated in Figure 6g and optically shown in Figure S27. In this configuration, the gas sensor was connected in series with the precharged flexible supercapacitor, and the response signals were wirelessly transmitted by the Bluetooth module (Figure 6h). When the PANI-based gas sensor is exposed to NH_3_, NH_3_ deprotonates the protonated PANI, imparting a more negative charge to the polymer and thereby increasing the resistance of the sensor. Upon re-exposure to ambient air, the PANI sensor can capture the hydrogen from the ammonium ion, and the resistance returns toward its baseline.^81^ This protonation–deprotonation process underpins the sensing mechanism of the PANI-based gas sensor. As shown in Figure 6i and Movie S2, the dynamic open-circuit voltage (OCV) response of the NH_3_ gas sensor exhibited high signal accuracy and stability across multiple exposure-recovery cycles. Each NH_3_ pulse induces a rapid OCV increase followed by a gradual relaxation toward the baseline in air. Notably, the response is most pronounced in the first cycle, while the peak amplitude progressively decreases in subsequent cycles, which is attributed to the incomplete desorption of NH_3_ from PANI and the partially irreversible deprotonation of protonated PANI in successive cycles. In addition, the voltage drop is limited to 9.2% of the initial voltage and is mainly ascribed to supercapacitor discharge, supporting the supercapacitor as a reliable power supply for wearable electronics. These results highlight the potential of the TSEL electrolyte for use in wearable energy storage devices.

### Generalized Strategy for Synthesizing Tough Ionogels

To further evaluate the generality of the EIPS strategy, both the polymer and ionic liquid species were varied. We replaced SEBS with SIS because of their comparable ionophobicity. As shown in Figure S28a, the TIEL-40E/10L ionogel exhibited the phase-separated structure induced by polarity-selective assembly, as evidenced by the EDS elemental mapping, wherein the distinct sulfur (S) and oxygen (O) signals originated from ionic species and the TPU phase. Meanwhile, the carbon-rich regions devoid of oxygen (O) were assigned to the SIS phase. This ionogel maintains excellent mechanical strength (8.63 MPa) and stretchability (1309%) (Figure S28b). Moreover, the calculated toughness of TIEL-40E/10L ionogel (45.2 MJ m^–3^) shows a slight increase compared with that of the TEL-40E/10L ionogel (43.8 MJ m^–3^), indicating a modest toughness enhancement associated with the phase-separated structure (Figure S28c).

Additionally, the 1-ethyl-3-methylimidazolium tetrafluoroborate (EMIM BF_4_) was used in place of EMIM TFSI. The resulting TPU/SEBS/EMIM BF_4_/LiTFSI ionogels retained the phase-separated morphology and exhibited enhanced mechanical properties. As shown in Figure S28d, the Raman spectra exhibit characteristic features of the TPU component and BF_4_^–^ from the EMIM BF_4_ ionic liquid. The morphology of TSEL-40B/10L ionogel differs significantly from that of the SEBS-free TEL-40B/10L ionogel, attributed to the incompatibility between the ionic components and the ionophobic SEBS phase (Figure S28e). Furthermore, the phase-separated structure of the TSEL-40B/10L ionogel could be confirmed by the EDS elemental mapping (Figure S28f). Similarly, the mapping reveals distinct sulfur (S) and oxygen (O) signals from the ionic components and the TPU phase, highlighting isolated ionic-rich regions, while carbon (C)-rich areas lacking oxygen correspond to the SEBS phase. Moreover, this phase-separated structure also contributes to the improved mechanical performance, wherein the TSEL-40B/10L ionogel achieved a strain of 830% and an ultimate strength of 8.64 MPa, compared to 450% strain and 6.27 MPa for the SEBS-free TEL-40B/10L ionogel (Figure S28g). The corresponding toughness reached 28.0 MJ m^–3^ for TSEL-40B/10L and 13.5 MJ m^–3^ for TEL-40B/10L (Figure S28h), confirming that the incorporation of ionophobic SEBS significantly enhances the mechanical properties of the ionogel. Additionally, in comparison with the above TSEL ionogel, the insufficient mechanical performance may result from the compatibility difference between the polymer matrices and the ionic components. These findings highlight the polarity-selectivity-induced phase-separation strategy as a versatile toughening method that enables straightforward customization of ionogel properties.

## Conclusions

In summary, we report a facile EIPS strategy to decouple ionic transport from mechanical robustness in ionogels without requiring complex syntheses and multistep processing. By exploiting the polarity contrast between the ionophilic TPU and ionophobic SEBS, together with the polarity-selective partitioning of ionic components, a well-organized bicontinuous architecture was spontaneously generated with a controlled solvent evaporation. In the phase-separated structure, the TPU-rich phase tends to concentrate the ionic species and establish continuous ion-transport pathways, whereas the SEBS-rich phase retains the original functions and serves as a mechanically dissipative scaffold that enables mechanical toughening without sacrificing ionic conductivity. These two phases work synergistically to strike a balance between ionic conductivity and mechanical robustness, endowing the ionogels with robust electrochemical properties, environmental stability, and recyclability. Furthermore, we extended the polarity-selective concept to another polymer-ionic liquid system and reproduced the same structure–property correlation, suggesting the generality of EIPS for designing high-performance ionogels. Multimodal ionic skins and wearable supercapacitors were successfully fabricated to demonstrate the application of our tough and stretchable ionogels. The EIPS strategy offers a simple and scalable route to preparing tough and stretchable ionogels, exhibiting great potential in the design of next-generation soft iontronics.

## Experimental Section

### Materials

1-Ethyl-3-methylimidazolium bis­(trifluoromethylsulfonyl)­imide was purchased from abcr GmbH. Lithium bis­(trifluoromethanesulfonyl)­imide and 1-ethyl-3-methylimidazolium tetrafluoroborate were provided by Sigma-Aldrich. TPU (Elastollan 1170A), SEBS (TUFTEC H1221), and SIS (D1111) were purchased from BASF, Asahi Kasei, and Kraton Corporation, respectively. THF and toluene were obtained from PENTA s.r.o. Polyaniline was purchased from Sigma-Aldrich. All chemicals were used as received. The polytetrafluoroethylene (PTFE) mold was sourced from Shenzhen Chaonai Plastic Hardware Materials Co., Ltd. Carbon cloth (thickness: ∼0.45 mm) was purchased from the Fuel Cell Store. Commercial polyimide sheet with a thickness of 0.15 mm was obtained from Dongguan Ronghui Insulation Materials Co., Ltd. Polyaniline used for the gas sensor was purchased from Sigma. The lithium polymer (Li-pol) battery was obtained from a local distributor of GE Electrics. The ESP32-C3 Super Mini development board was purchased for signal acquisition and wireless communication.

### Preparation of Polymer Matrices

The TPU and SEBS were separately dissolved in THF and toluene at a weight ratio of 1:6, corresponding to polymer concentrations of 14.29 wt %. The solutions were homogenized using a rotary shaker (Heidolph Rotamax 120, 150 rpm) for 8 h. Subsequently, the TPU and SEBS solutions were combined at the desired weight ratios and magnetically stirred vigorously at 500 rpm and 40 °C for 4 h in a sealed glass bottle to prevent premature solvent evaporation using a magnetic stirrer with a temperature-controlled hot plate. The resulting homogeneous mixture was cast into a PTFE mold with a partially covered lid to mitigate pore formation from rapid solvent evaporation and dried under ambient conditions for 3–4 days, resulting in a uniform and stretchable polymer film.

### Preparation of Various Ionogels

TPU and SEBS were separately dissolved in THF and toluene, respectively, at a weight ratio of 1:6, yielding polymer solutions with concentrations of 14.29 wt %. The two solutions were then combined at a TPU:SEBS mass ratio of 7:3 in a sealed glass bottle and stirred thoroughly to form a homogeneous polymer solution as mentioned above. Separately, EMIM TFSI was added at 20 wt %, 30 wt %, 40 wt %, and 50 wt %, together with LiTFSI at a fixed 10 wt %, with all weight percentages calculated relative to the total mass of the final mixture, including polymers without solvent and ionic components. EMIM TFSI and LiTFSI were first premixed and fully dissolved using an ultrasonic bath for 10 min. Then, the polymer solution was subsequently added to the ionic liquid-salt mixture and magnetically stirred at 500 rpm and 40 °C for 4 h to ensure complete mixing and dispersion using a magnetic stirrer with a temperature-controlled hot plate. To minimize premature solvent evaporation (given the high volatility of THF and toluene) and reduce exposure to moisture, all solution preparation steps were conducted in sealed glass containers, and the total processing time before casting was kept short.

The resultant mixture was cast into the partially covered PTFE mold, allowing for slow and controlled solvent evaporation under ambient conditions over 3–4 days. Gradual solvent removal minimized phase competition during EIPS and prevented the formation of pores, obtaining uniform, optically transparent ionogel films (thickness: 0.15 ± 0.02 mm). For comparison, control ionogels containing only LiTFSI (without EMIM TFSI), SEBS-free ionogels, as well as those prepared by replacing EMIM TFSI and SEBS with EMIM BF_4_ and SIS, respectively, to promote the generalization of the strategy, were fabricated following the same procedure.

### Materials Characterizations

Bruker Vertex 70v was used to record the Fourier transform infrared spectrometer (FTIR) spectroscopy of the samples (600–4000 cm^–1^, resolution at 2 cm^–1^). Scanning electron microscopy (SEM) (MIRA3 XMU) equipped with an energy-dispersive spectrometer (EDS) was used to characterize the morphology of the ionogels (5 kV for morphology; 20 kV for elemental mapping). For SEM observation under strain, the rectangular samples were first stretched uniaxially to 200% strain and mounted on the SEM stub in the stretched state using conductive carbon tape. To further minimize relaxation during transfer and imaging, the two ends of the samples were additionally secured with carbon tape. Before characterization, the samples were sputter-coated with a thin layer of gold using a sputter coater (Leica EM ACE600). SEM images of the recovered state were collected after release from 200% strain. Atomic force microscopy (AFM) and Kelvin probe force microscopy (KPFM) measurements were carried out using Bruker Dimension Icon AFM. The X-ray diffraction (XRD) profile of the ionogels was examined by an X-ray powder diffractometer, Rigaku SmartLab 3 kW (RIGAKU3). Differential scanning calorimetry (DSC) of the samples was assessed via a DSC-Discovery (TA Instruments) under a nitrogen atmosphere at a scan rate of 2 °C min^–1^. Thermal stability analysis was conducted on a thermogravimetric analyzer named TGA-DISCOVERY from TA Instruments over the temperature range of 50–600 °C in a nitrogen atmosphere with a heating rate of 20 °C min^–1^. All the samples were carried out at 40 °C under a vacuum of approximately 200 mbar for 6 h to remove the residual solvent and moisture. Dynamic mechanical analysis (DMA) was performed on an RSA-G2 dynamic mechanical analyzer (TA Instruments, USA) in tensile mode at a frequency of 1 Hz under a nitrogen atmosphere to evaluate the viscoelastic properties of the TSEL ionogels. The rectangular-shaped samples with dimensions of approximately 30 × 10 × 0.2 mm^3^ were measured in the temperature range from −75 to 100 °C at a heating rate of 2 °C min^–1^. Transmittance of the ionogels with a thickness of 0.3 mm was carried out using the V-730 UV–vis spectrophotometer under the wavelength for testing ranging from 800 to 400 nm. The temperature distribution on the samples was recorded using the infrared (IR) camera (Seek Thermal, California, USA). During the humidity test, the ionogel was placed in a closed chamber, where the dry air and humid air with different relative humidity levels were switched by a lab-developed gas control system.^82^ The resistance change under combined humidity and temperature was measured in real-time using a multimeter. For the performance testing in harsh environments, the performances of TSEL ionogels were recorded by placing them in a vacuum oven, underwater (deionized water), and a refrigerator (−20 °C), respectively. All measurements were performed under ambient laboratory conditions of 23 ± 2 °C and 30 ± 5% relative humidity. The weight retention (*WR*) capacity under different temperatures was calculated using eq 1:

1 $$WR\left(\%\right)=\left(W_{n}/W_{0}\right)\times 100\%$$

where *W*_0_ is the initial weight of the ionogel, and *W*_*n*_ is the weight at different time points.

The relative mass loss (Δ*W*_*t*_) of the samples immersed in deionized water was defined by eq 2:

2 $$\Delta W_{t}\left(\%\right)=\frac{W_{t}-W_{0}}{W_{0}}\times 100\%$$

where *W*_0_ is the initial weight of the ionogel, and *W*_*t*_ is the weight of the ionogels after being immersed in deionized water for specified time points, followed by blotting with filter paper to remove excess liquid.

The ionic conductivities (σ) of the prepared samples were measured by an impedance spectrometer (Novocontrol Alpha-A modular) with a frequency range from 0.1 to 1 MHz, and then were calculated using eq 3:

3 $$\sigma =\frac{L}{R_{b}A}$$

where *R*_b_ is the bulk resistance from the Nyquist plot, *L* is the sample thickness, and *A* is the area of the ionogels. Except for the temperature-dependent measurements, ionic conductivity was measured under ambient conditions with a relative humidity of 30 ± 5% and a temperature of 25 °C.

### Mechanical Testing

The mechanical tests of the ionogels were conducted by the ZwickRoell Z010 AllroundLine system. Tensile experiments were conducted at a strain rate of 30 mm min^–1^ for both stretching and relaxation rates at room temperature (23 ± 2 °C) and ambient humidity (30 ± 5%). Dumbbell-shaped samples followed GB/T 528 Type 2 dimensions. The thickness of the samples for tensile tests was 0.15 ± 0.02 mm. Young’s modulus was calculated from the slope of the initial linear region of the stress–strain curves. Toughness was determined by integrating the area under the stress–strain curves up to fracture. Samples for the compression test were prepared in dimensions of 16 × 16 × 1.2 mm^3^. The compression rate was at 1 mm min^–1^. Samples for pure shear tests had a thickness of 0.2 mm and a width of 15 mm, with a fixed gauge length of 4 mm. The notched samples were precut with a crack length of 7 mm. Fracture tests were carried out at a strain rate of 0.001 s^–1^. The fracture energy of the ionogels was calculated using eq 4:

4 $$\Gamma =H\omega \left(\lambda_{c}\right)$$

where λ_c_ is the critical stretch of the crack propagation in the notched samples, ω­(λ_c_) is the area under the stress–strain curves of the unnotched samples over the critical stretch range, and *H* is the gauge length.

### The Fabrication and Measurement of I-Skins

I-skins based on the TSEL ionogels used for strain sensing were prepared with dimensions of 50.0 × 8.0 × 0.15 mm^3^. The size of the ionogels that were used for pressure and temperature sensing was 16 × 16 × 1.2 mm^3^. Two copper electrodes were then attached to both ends of the TSEL ionogels for testing. The resistance changes of the I-skin based on the ionogels were measured using a PGSTAT204 electrochemical workstation (Metrohm Autolab), with data acquisition and analysis performed via NOVA 2.1 software, in combination with the ZwickRoell Z010 AllroundLine mechanical testing system. In specific, the I-skins were affixed to the finger, wrist, elbow, and knee using the insulated tape (3M VHB 9473) to monitor human motions.

### Fabrication of Wearable Supercapacitors

A few drops of *N*-methyl-2-pyrrolidone were mixed with the active material, consisting of 90 wt % activated carbon and 10 wt % carbon black. Without the use of any binders, the resulting mixture was applied onto the flexible carbon cloth using the doctor blade method. The coated electrode was then dried in a vacuum oven overnight (12 h). The symmetric flexible supercapacitor was assembled by stacking a five-layer structure consisting of polyimide film/activated carbon cloth/TSEL-40E/10L electrolyte/activated carbon cloth/polyimide film. The assembled device was precharged using an Autolab electrochemical workstation before use.

### Electrochemical Analysis of Flexible Supercapacitors

Electrochemical measurements were performed using a PGSTAT 204 electrochemical workstation (Autolab) with data acquisition and analysis carried out via Nova 2.1 software. The electrochemical performance of the fabricated symmetric flexible supercapacitor was evaluated using cyclic voltammetry (CV) and galvanostatic charge–discharge (GCD) tests. CV and GCD profiles were recorded at various scan rates and current densities. The specific capacitance (*C*_sp_, F g^–1^) was calculated using eq 5:

5 $$C_{sp}=\frac{I\Delta t}{m\Delta V}$$

where *I* is the discharge current (A), Δ*t* is the discharging time (s), *m* is the total mass of the two electrodes (g), and Δ*V* is the potential window (V). All electrochemical measurements were conducted at room temperature (23 ± 2 °C) and ambient humidity (30 ± 5%).

### Wearable Supercapacitor-Integrated Ammonia Gas Monitoring System

The ammonia gas sensor was fabricated by drop-casting polyaniline onto an Al_2_O_3_ substrate patterned with Au interdigitated electrodes, followed by drying in a vacuum oven at 60 °C for 2 h. The sensor device was powered by the fabricated supercapacitor. The data were recorded and wirelessly transmitted to the LabVIEW-based program via an ESP32-C3 module with Bluetooth functionality, which was programmed via Arduino and powered by a Li-Pol battery. The initial concentration of ammonia gas supplied to the system was 200 ppm.

## Supplementary Material







## Notes

Chemical structures of the materials used; Optical transmittance spectra of TSEL ionogels; Property details of various polymer matrices, including DSC, mechanical performance, and DMA; Property details of EMIM TFSI-free ionogels, including FTIR, electrical, and mechanical performance; Characterization and mechanical properties of SEBS-free ionogels; Characterization of TSEL ionogels, including Raman, DSC, FTIR, XRD, toughness, electrochemical impedance spectroscopy, and ionic conductivity; Characterization of the TSEL-40E/10L ionogel including mechanical performance, surface morphology, electrical performance, and environmental tolerance; Sensing performance of the I-skin; CV profiles of the flexible supercapacitor; Wearable gas-sensing demonstration; Generality of the EIPS strategy; Performance comparison of TSEL ionogel with previously reported ionogels (Tables S1 and S2); XPS atomic percentage analysis (Table S3); Comparison with representative TPU-based ionogels (Table S4) (PDF)
Underwater strain sensing using the TSEL ionogel-based ionotronic skin (MP4)
Wireless monitoring of ammonia using a wearable supercapacitor-integrated system (MP4)
