# Supplementary material for: Polarity-Selective Assembly Enables Tough and Stretchable Ionogels for Wearable Electronics
Source: ACS Nano. 2026 Jun 23;20(26):18700–18. doi: 10.1021/acsnano.6c02932 (PMC13348173; doi:10.1021/acsnano.6c02932)
Supplement: Supplementary file 1 [file nn6c02932_si_001.pdf]

Supporting Information

Polarity-Selective Assembly Enables Tough and Stretchable Ionogels for Wearable Electronics

Hongbo Fu[1], Xia Peng[1], Kaaviah Manoharan[1], Xiaohui Ju[1], Sanjay Kumar[1], Martin Pumera*[1,2,3]

[1]Future Energy and Innovation Laboratory, Central European Institute of Technology, Brno University of Technology, Purkyňova 123, Brno, 612 00, Czech Republic

[2]Advanced Nanorobots & Multiscale Robotics Laboratory, Faculty of Electrical Engineering and Computer Science, VSB - Technical University of Ostrava, 17. listopadu 2172/15, 70800 Ostrava, Czech Republic

[3]Department of Medical Research, China Medical University Hospital, China Medical University, No. 91 Hsueh-Shih Road, Taichung 40402, Taiwan

This file includes:

Figure S1 to Figure S28.

Tables S1 to S4.

SI References.

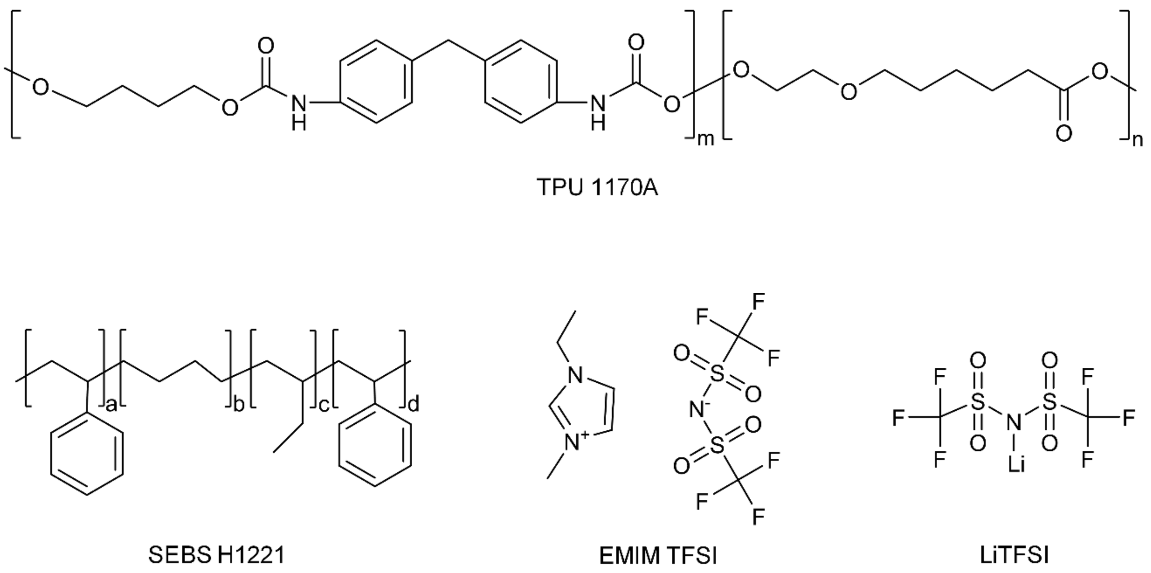

Figure S1. Chemical structures of used materials.

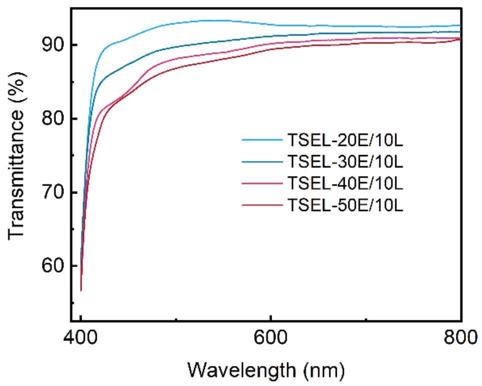

Figure S2. Transmittance spectra of the TSEL ionogels in the visible wavelength range.

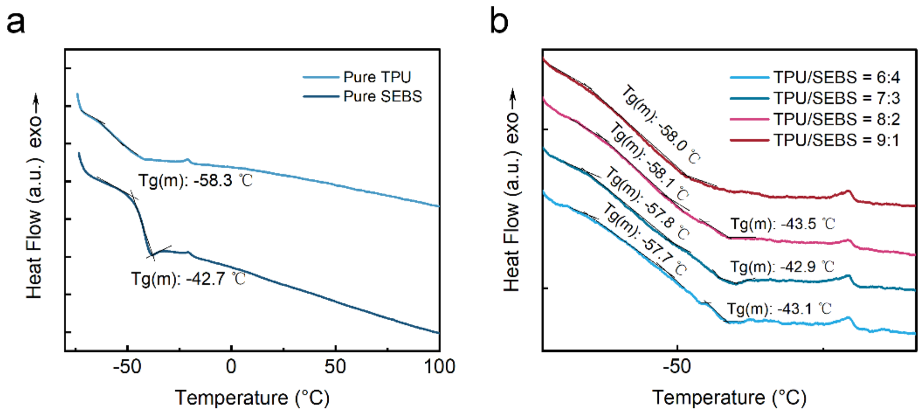

Figure S3. (a) Differential scanning calorimetry (DSC) traces of pure TPU and pure SEBS. **(b)** DSC traces of polymer matrices with different SEBS contents, focusing on the low-temperature region.

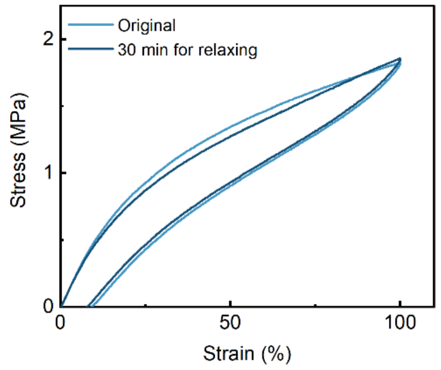

Figure S4. Stress–strain curves of TPU/SEBS = 7:3 matrix subjected to cyclic loading at a strain of 100% with a 30-minute recovery interval.

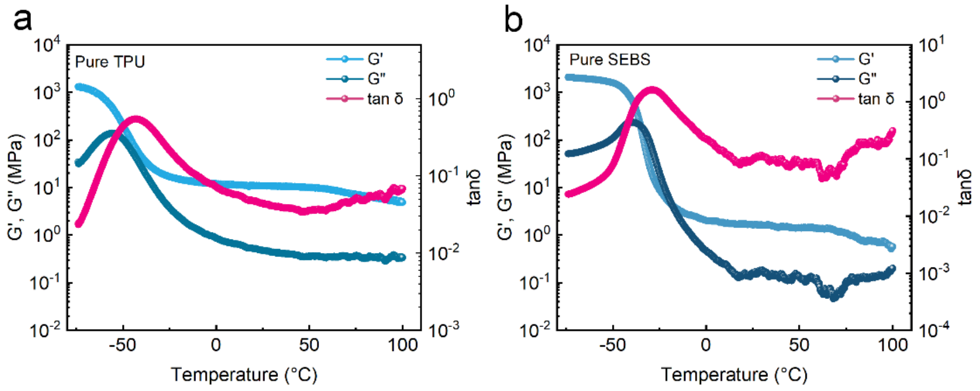

Figure S5. Dynamic mechanical analysis (DMA) temperature sweep curves of **(a)** pure TPU and **(b)** pure SEBS.

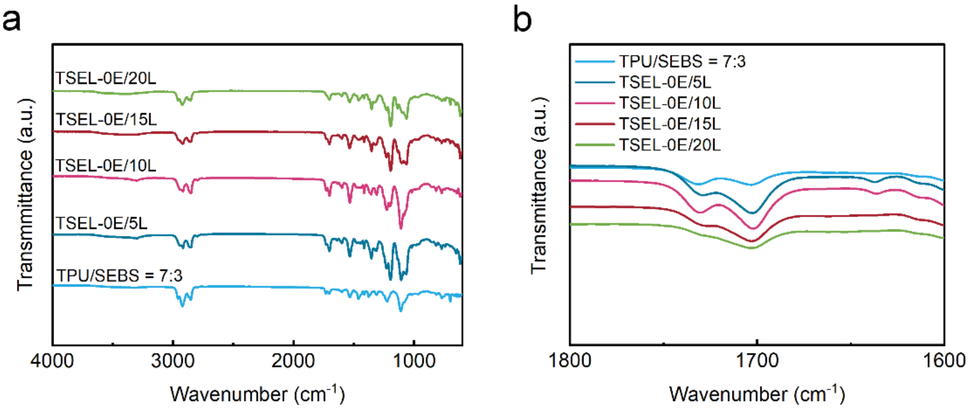

Figure S6. (a) Fourier-transform infrared spectroscopy (FTIR) spectra of the ionogels with varying LiTFSI content. **(b)** FTIR spectra of the ionogels focusing on the C=O stretching vibration region.

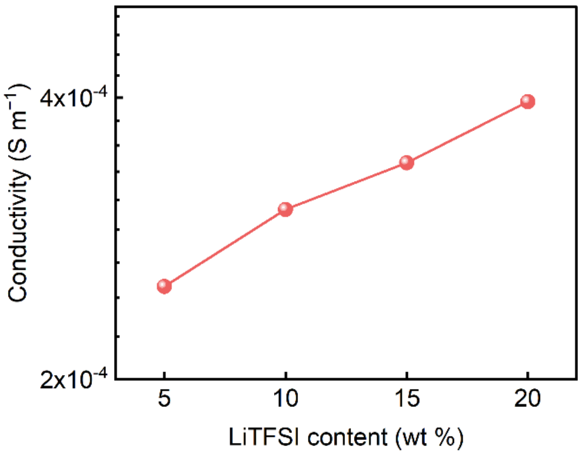

Figure S7. Ionic conductivity of EMIM TFSI-free ionogels with varying LiTFSI content.

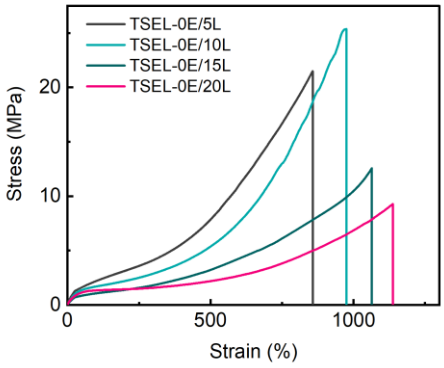

Figure S8. Stress–strain curves of EMIM TFSI-free ionogels with different LiTFSI contents.

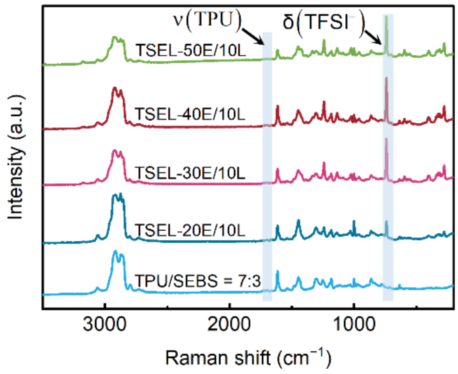

Figure S9. Raman spectra of TPU/SEBS = 7:3 matrix and the TSEL ionogels; gray frames highlight characteristic peaks associated with the TPU backbone and the $TFSI^-$ anion.

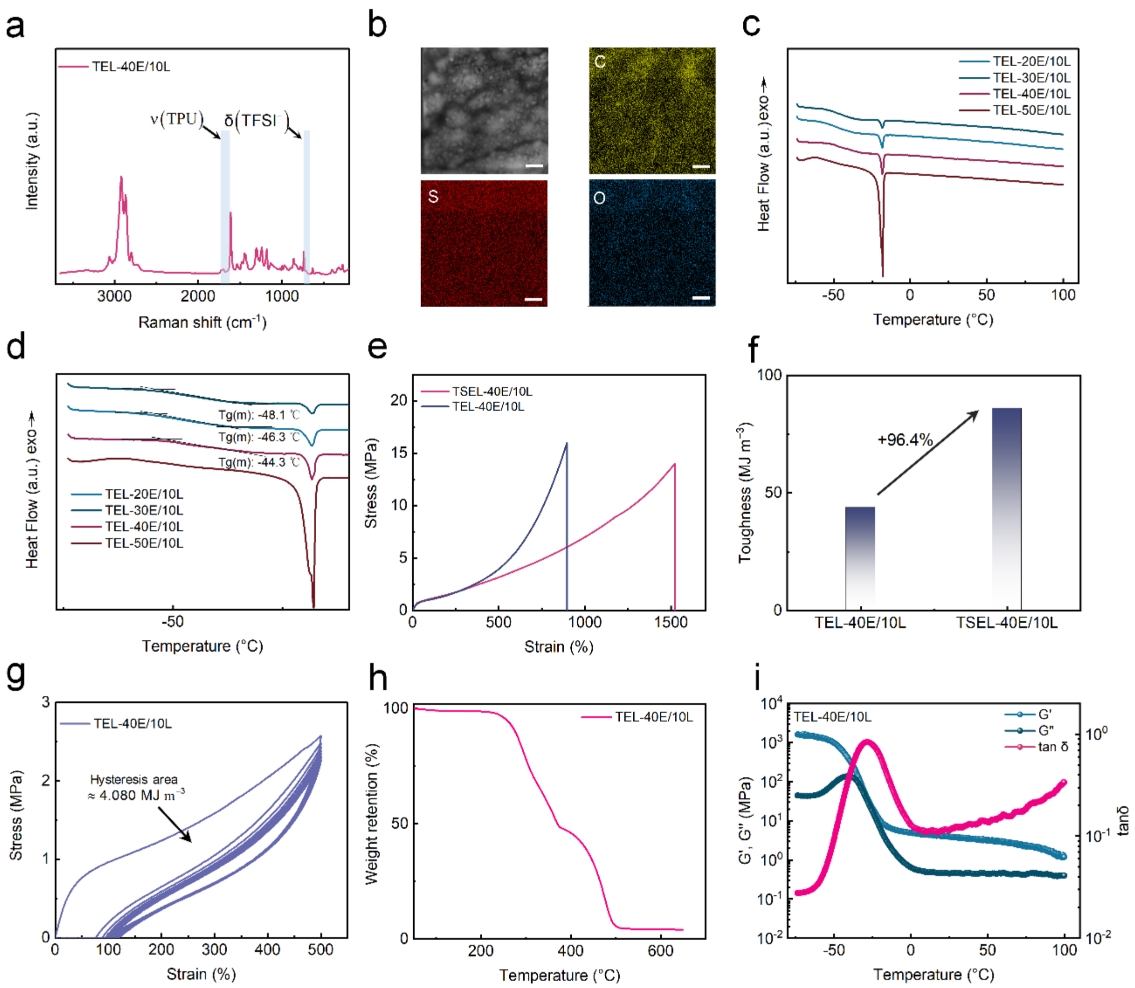

Figure S10. Characterization and mechanical properties of SEBS-free ionogels. (a) Raman spectrum of the SEBS-free TEL-40E/10L ionogel. **(b)** Scanning electron microscopy (SEM) image and corresponding energy-dispersive X-ray spectroscopy (EDS) elemental maps of carbon (C), sulfur (S), and oxygen (O) for the SEBS-free TEL-40E/10L ionogel. Scale bars: 20 μm. **(c)** Differential scanning calorimetry (DSC) traces of TEL ionogels with different EMIM TFSI content. **(d)** DSC traces of the TEL ionogels, focusing on the low-temperature region. **(e)** Stress–strain curves of TSEL-40E/10L ionogel and SEBS-free TEL-40E/10L ionogel. **(f)** Comparison of toughness enhancements for TSEL-40E/10L ionogel and SEBS-free TEL-40E/10L ionogel. **(g)** Consecutive cyclic tensile curves of TEL-40E/10L ionogel at 500% strain. **(h)** Thermogravimetric analysis (TGA) curve of TEL-40E/10L ionogel. **(i)** DMA temperature sweep curves of SEBS-free TEL-40E/10L ionogel.

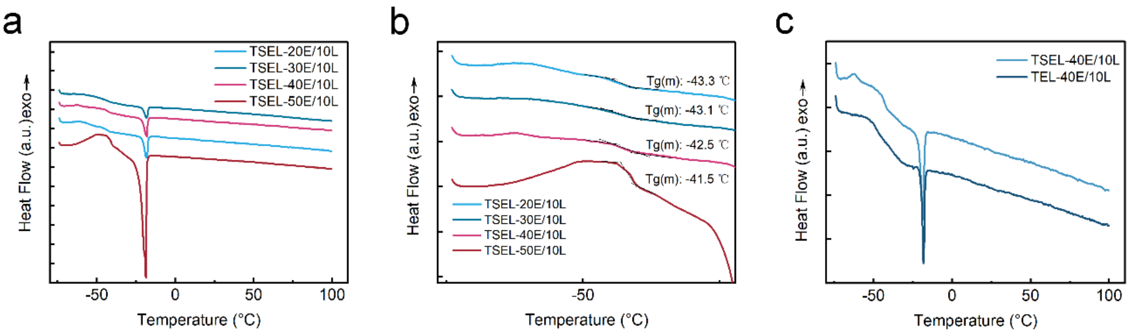

Figure S11. (a) DSC traces of TSEL ionogels with different EMIM TFSI content. **(b)** DSC traces of the TSEL ionogels, focusing on the low-temperature region. **(c)** Comparative DSC traces of TSEL-40E/10L and TEL-40E/10L ionogels.

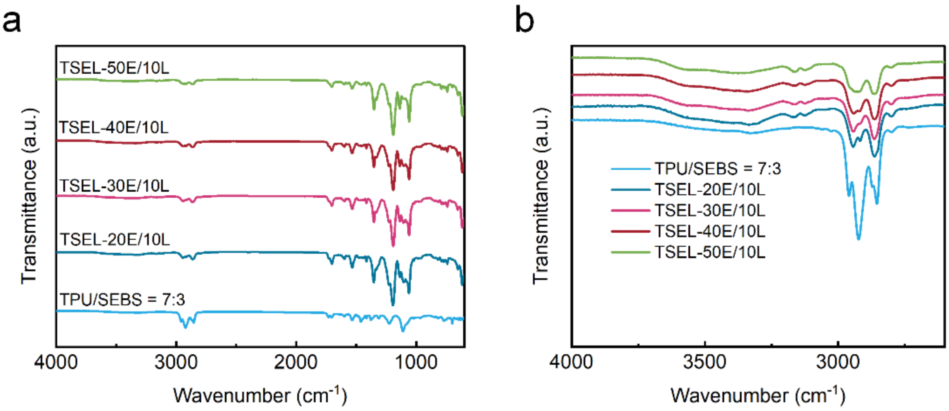

Figure S12. (a) FTIR spectra of ionogels with various EMIM TFSI content. **(b)** Wavenumber region of 2600–4000 cm^{-1}.

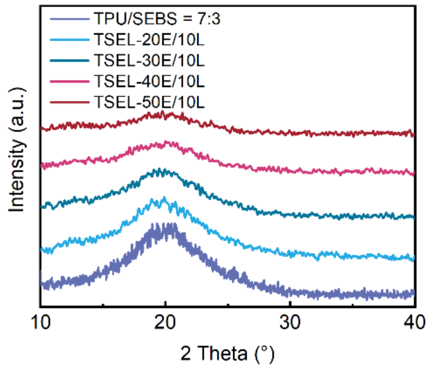

Figure S13. X-ray diffraction (XRD) profile of TPU/SEBS = 7:3 and TSEL ionogels with varying EMIM TFSI content.

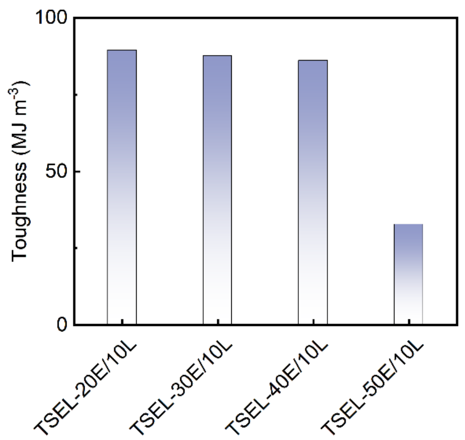

Figure S14. Toughness of TSEL ionogels with varying EMIM TFSI content.

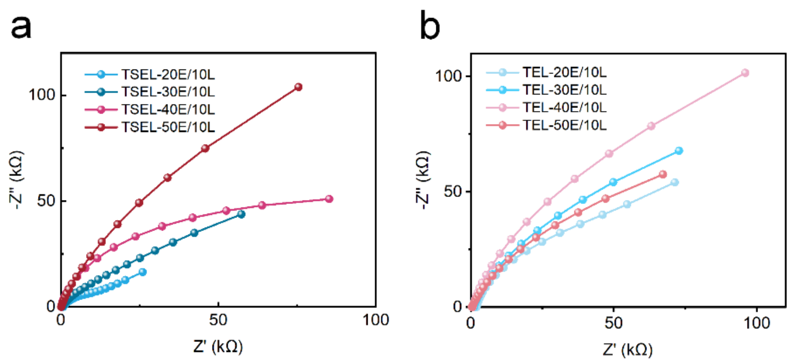

Figure S15. Representative Nyquist plots of **(a)** TSEL ionogels and **(b)** SEBS-free TEL ionogels at different EMIM TFSI contents.

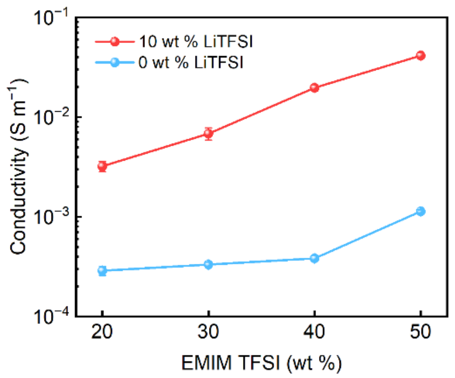

Figure S16. Effect of LiTFSI incorporation on the ionic conductivity of TSEL ionogels with different EMIM TFSI contents.

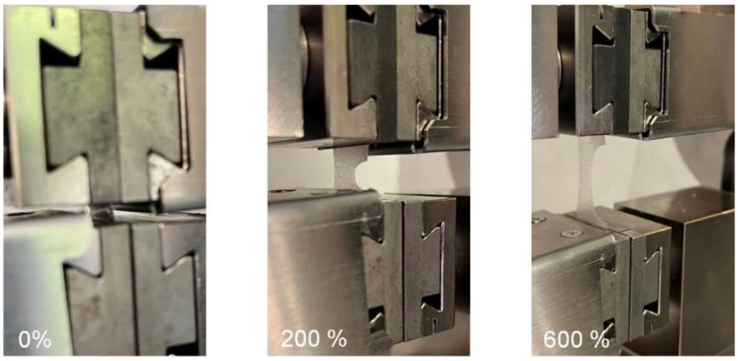

Figure S17. Pure–shear deformation of the TSEL-40E/10L ionogel at representative strains (0%, 200%, and 600%).

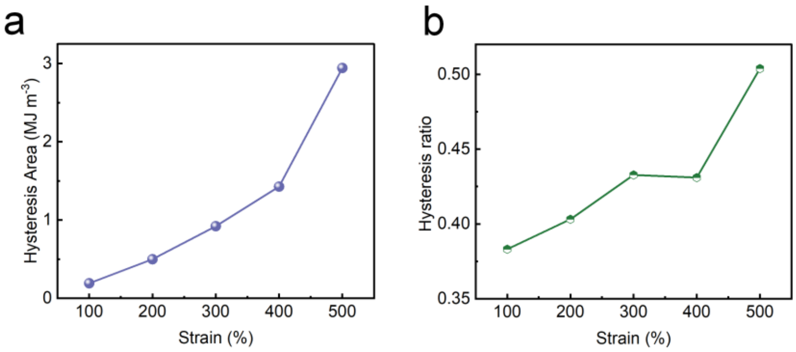

Figure S18. (a) Hysteresis energy and **(b)** hysteresis ratio as a function of applied strain (100−500%) for the TSEL-40E/10L ionogel.

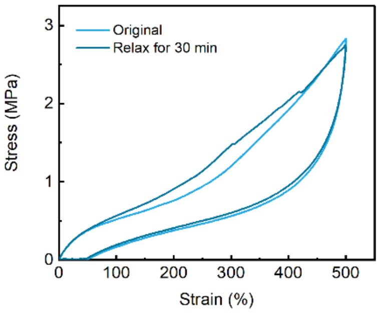

Figure S19. Stress–strain curves of the TSEL-40E/10L ionogel during a loading−unloading cycle to 500% strain, comparing the original response and the response after 30 min recovery.

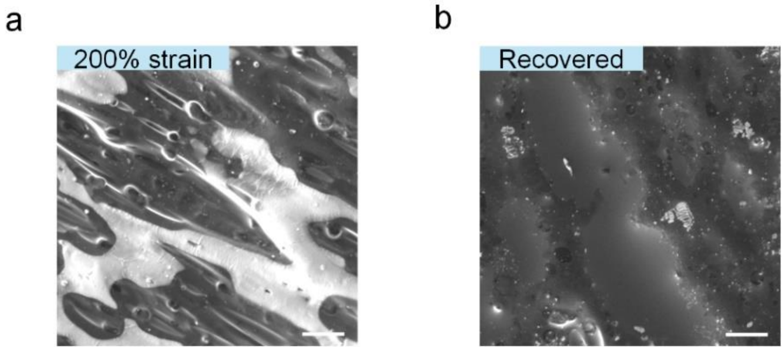

Figure S20. (a) Section view SEM images of TSEL-40E/10L ionogel under the strain of 200% and **(b)** recovered from 200% strain. Scale bars: 20 μm.

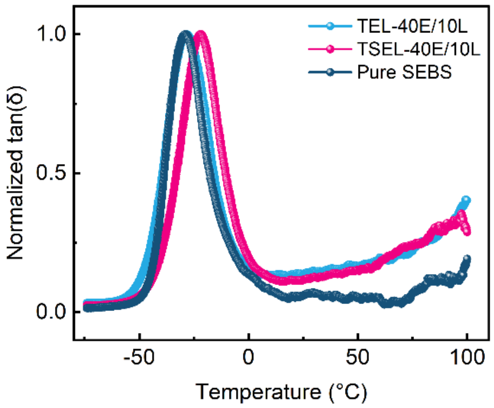

Figure S21. Normalized tan δ as a function of temperature for TEL-40E/10L, TSEL-40E/10L, and pure SEBS.

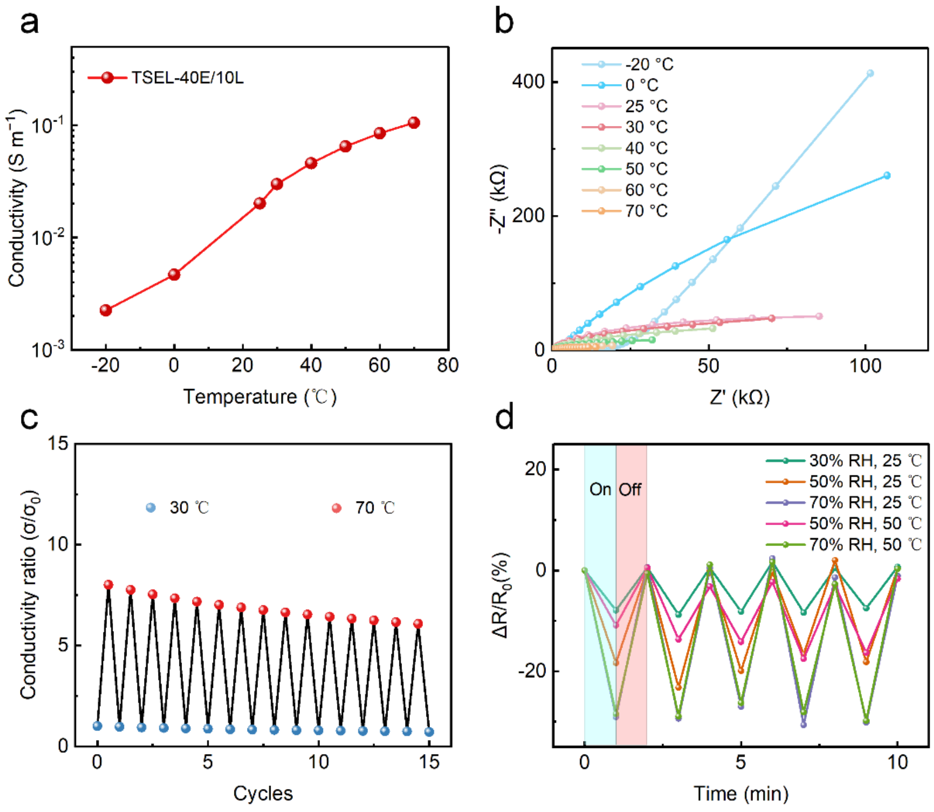

Figure S22. Electrical performance and environmental tolerance of the TSEL-40E/10L ionogel. (a) Ionic conductivity of the ionogel as a function of temperature. **(b)** Nyquist plots of the ionogel at different temperatures. **(c)** Ionic conductivity changes (σ/σ_0; σ_0: initial ionic conductivity, σ: ionic conductivity measured during temperature cycling) of the ionogel over the heating–cooling cycles between 30 and 70 °C. **(d)** Relative resistance changes of the ionogel under cyclic humidity and temperature variations.

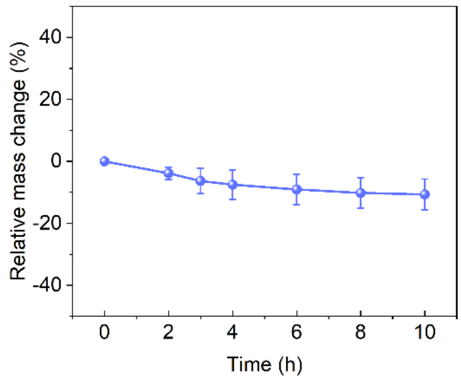

Figure S23. Relative mass change of TSEL-40E/10L as a function of immersion time in deionized water.

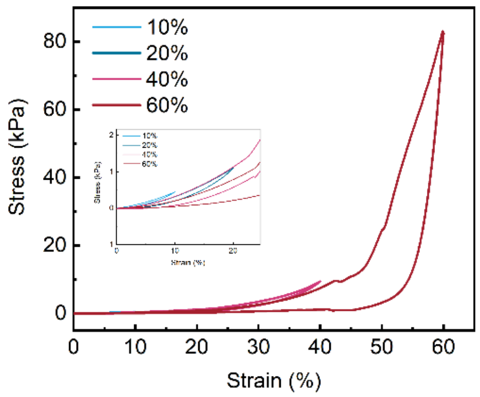

Figure S24. Compression tests of the TSEL-40E/10L ionogel under different compression strains (10% to 60%). The inset shows the magnified view of the curves in the low-strain region (0–20%).

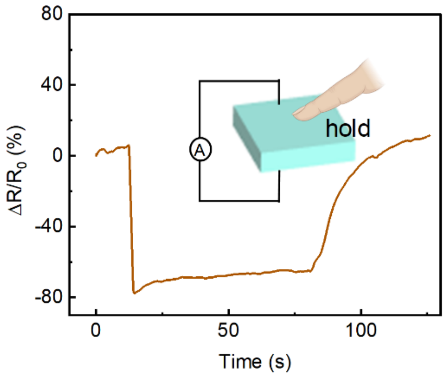

Figure S25. Relative resistance changes of the I-skin in response to a stepwise finger compression.

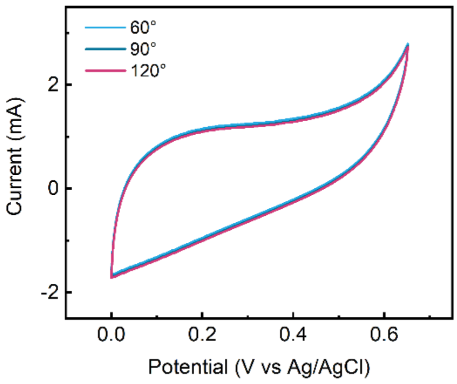

Figure S26. Cyclic voltammetry curves under different bending angles (60°, 90°, and 120°) at a scan rate of 50 mV s^{-1}.

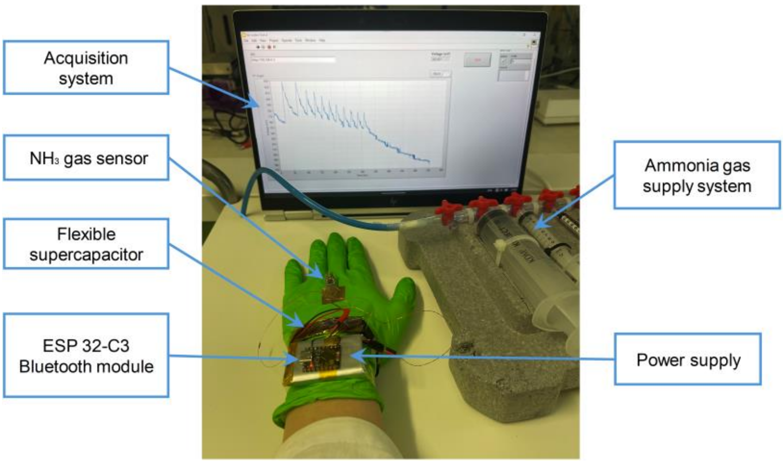

Figure S27. Wearable wireless ammonia gas sensing system integrated with the ionogel-based wearable supercapacitor.

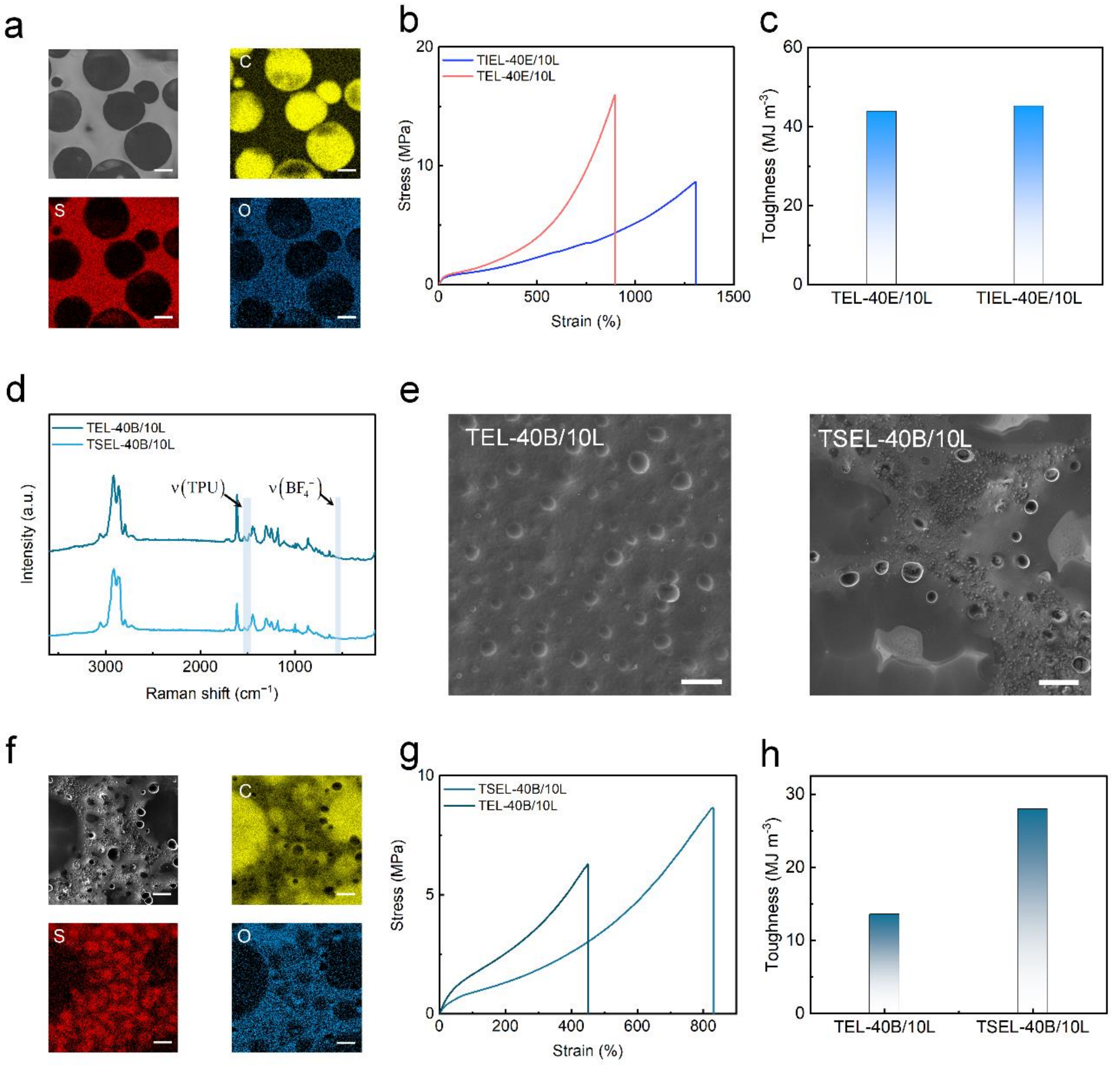

Figure S28. Generality of the EIPS strategy in ionogels with varied polymer and ionic liquid species. (a) SEM image and corresponding EDS elemental maps of carbon (C),

sulfur (S), and oxygen (O) for the TIEL-40E/10L ionogel. Scale bars: 20 μm. **(b)** Stress–strain curves of TIEL-40E/10L and TEL-40E/10L ionogels. **(c)** Toughness comparison between the TIEL-40E/10L and TEL-40E/10L ionogels. **(d)** Raman spectra of the TSEL-40B/10L and SEBS-free TPU/EMIM BF_4/LiTFSI ionogel (TEL-40B/10L); gray frames highlight characteristic peaks associated with the TPU backbone and the BF_4^- anion. **(e)** SEM images of TSEL-40B/10L and the SEBS-free TEL-40B/10L ionogels. Scale bars: 20 μm. **(f)** EDS elemental maps of sulfur (S), oxygen (O), and carbon (C) for the TSEL-40B/10L ionogel. Scale bars: 20 μm. **(g)** Stress–strain curves of TSEL-40B/10L and TEL-40B/10L ionogels. **(h)** Toughness comparison between the TSEL-40B/10L ionogel and TEL-40B/10L ionogel.

Table S1. A performance comparison of TSEL ionogel with the previously reported ionogels prepared by different methods

Material system	Methods	Maximum strain	Strength [MPa]	Toughness [MJ m^{-3}]	Recyclability	Ref.
PDADMAC/ PNHEAA/glycerol	Polymerization	513%	6.3	20.3	NR	1
ACMO/[EMIM] [DCA]/[N_{4111}] [NTf_2]	Polymerization	980%	0.97	4.9	NR	2
HEAA/SPP/ChCl-EG	Polymerization	948.4%	3.57	25.3	NR	3
IBA/HFBA/ [AETA][TFSI]/ /[EMIM][TFSI]	Polymerization	1504%	1.13	9.18	Yes	4
P(AA-co-MEA)/ [EMIM][NTf_2]	Polymerization	716%	0.28	1.01	NR	5
PAM/[EMIM] $(EtO)_2PO_2$	Polymerization	747.1%	6.42	18.08	NR	6
P(TFEA-co-AAm)/ [EMIM] [TFSI]	Polymerization	1828%	0.55	3.66	Yes	7
P([MATAC][TFSI]-co-AM)	Polymerization	570%	2.75	9.58	NR	8
RM257/EDDET/ PETMP/[BMIM][PF_6]	Polymerization	2700%	4.7	56.9	NR	9
P(TA-co-AM)/ [EMIM][ESO_4]	Polymerization	312%	17.1	40.4	NR	10
PVA/[BMI][TFSI]	Solvent exchange	2600%	9.9	93.42	Yes	11
PEGDA/PETA/BTCA/ PIL-BF_4/ED	Solvent exchange	1390%	2.28	9	NR	12

**Table S1** continued						
PU/[DEIM][TFSI]	Solvent evaporation	327%	1.54	2.5	Yes	13
poly(MA-r-4HS)-b-PtBS/ [EMIM][TFSI]	Solvent evaporation	720%	0.65	2.479	Yes	14
DOU-CPU /[EMIM][TFSI]	Solvent evaporation	834%	0.76	2.39	Yes	15
TPU/SEBS/[EMIM][TFSI] /LiTFSI	Solvent evaporation	1522%	12.8	86.08	Yes	**This work**

Note: NR=Not reported

Table S2. A mechanical performance comparison of TSEL ionogel with the various reported ionogels

Material system	types	Maximum strain	Strength [MPa]	Toughness [MJ m^{-3}]	Ref.
P(AM-co-DMAAm)/[EMIM][Br]	Phase-separated ionogel	404.4%	8.94	21.94	16
		1319.05%	0.113	1.01	
PVA/[AMIM][Cl]/[EMIM] [$EtSO_4$]	Phase-separated ionogel	332%	0.0091	0.015	17
		582%	0.0262	0.067	
		1030%	0.0497	0.210	
P(NIPAM-co-AA)/[EMIM][$EtSO_4$]	Phase-separated ionogel	473%	11.73	28.09	18
PAM/ureido-functionalized imidazolium iodide IL	Phase-separated ionogel	685.49%	0.35	7.57	19
		180%	5.19	1.1	
P(AA-co-HFBA)/PEO/ [BMIM][BF_4]	Phase-separated ionogel	380%	NR	1.3	20
		2500%	NR	7.4	
HPC/LA/AA/[C_2VIm][Br]	Phase-separated ionogel	4222%	NR	1.03	21

Table S2 continued

QMFB/[Ch][TFSI]	Phase-separated ionogel	1460%<br>1030%<br>290%	0.43<br>1.4<br>3.66	3<br>6.8<br>6.91	22
P(AM-co-AA-co-HEA)/[EMIM][EtSO$_4$]/[BMIM][OAc]	Phase-separated ionogel	960%<br>975%<br>810%	1.41<br>3.29<br>3.57	6.55<br>13.15<br>12.09	23
PEO/LiTFSI/[EMIM][Cl]	Phase-separated ionogel	2500%	NR	2.6	24
Poly(NIPAm-co-AAc)/menthol-decanoic acid DES	Phase-separated ionogel	520%	NR	14.3	25
PDMAA/[EMIM][TFSI]/[EMIM][OAc]	Tough ionogel	1300%	16.9	96.86	26
ZPU/[Omim][Cl]	Tough ionogel	1075%<br>1310%	22.4<br>8.8	84.2<br>33.0	27
PAA-F/H2O/[BMIM][BF$_4$]	Composite ionogel	5726%	0.73	4.4	28
TPU/SEBS/[EMIM][TFSI]/LiTFSI	Phase-separated ionogel	1188%<br>1396%<br>1522%	22.01<br>17.8<br>12.8	89.4<br>87.63<br>86.08	**This work**

Note: NR=Not reported

Table S3. XPS atomic percentage analysis based on the survey spectra

Samples	C [%]	O [%]	N [%]	F [%]	S [%]	F/S
TSEL-40E/10L-day 0	70.6	26.6	0	2.2	0.5	4.4
TEL-40E/10L-day 0	40.2	16.8	10.8	24.2	8	3
TSEL-40E/10L-day 7	80.3	16.9	0	1.8	1	1.7
TEL-40E/10L-day 7	39.6	17.4	11.2	24	7.8	3.1

Table S4. Comparison of electromechanical performance and durability of the TSEL ionogel with representative TPU-based ionogels

Material system	Ionic conductivity [S m^{-1}]	Strength [MPa]	Toughness [MJ m^{-3}]	Mechanical durability	Environmental stability	Ref.
TPU/SiO_2-[BMP][NTf_2]	2.5×10^{-4}	3.43	NR	50% strain for 1000 s	water-immersion stability	29
BP/PVP/TPU/[BMIM][PF_6]	NR	0.59	NR	30% strain, 1000 cycles	-35 °C for up to 15 d	30
TPU/[BMIM][PF_6]/HNTs	8.8×10^{-1}	8.04	NR	40% strain, 5625 cycles	NR	31
TPU/CNTs/[BMP][NTf_2]	6.4×10^{-4}	6.6	NR	20% strain, 1000 cycles	water-immersion stability	32
TPU/BP /[BMIM][Cl]	NR	17.18 ± 1.35	13.14 ± 1.38	30% strain, 1000 cycles	humidity resistance	33
TPU/BP /[BMIM][PF_6]	NR	16.91	14.85	10% strain, 1000 cycles	humidity resistance	34
TPU/SEBS/[EMIM][TFSI]/ LiTFSI	1.97×10^{-2}	12.8	86.1	100% strain, 500 cycles	ambient stability; wide-temperature operation; thermal/humidity cycling; water-immersion stability	**This work**

Note: NR=Not reported

References

[1] Ge, G.; Zhang, Y.; Xiao, X.; Gong, Y.; Liu, C.; Lyu, C.; Ong, W. L.; Ho, G. W.; Yang, Z.; Huang, W., Rapidly gelling, highly adhesive, and mechanically robust ionogels for stretchable and wireless electronics. *Adv. Funct. Mater.* **2024,** *34* (21), 2310963, DOI: 10.1002/adfm.202310963

[2] Tie, J.; Mao, Z.; Zhang, L.; Zhong, Y.; Xu, H., Strong and ultratough ionogel enabled by ingenious combined ionic liquids induced microphase separation. *Adv. Funct. Mater.* **2023,** *33* (52), 2307367, DOI: 10.1002/adfm.202307367

[3] Cong, Z.; Cui, Z.; Liu, C.; Wang, J.; Huo, X.; Xu, J.; Niu, J., High strength and tough ionogels with bicontinuous phase network structure induced by electrostatic adsorption triggered microphase separation. *Adv. Funct. Mater.* **2024,** *34* (52), 2410588, DOI: 10.1002/adfm.202410588

[4] Zhou, R.; Jin, Y.; Li, Y.; Jin, H.; Zeng, W.; Mei, J.; Liu, Y., In-situ phase separation constructing robust hydrophobic ionogels with multifunction. *Chem. Eng. J.* **2023,** *476*, 146840, DOI: 10.1016/j.cej.2023.146840

[5] Zhang, J.; Yin, J.; Li, N.; Liu, H.; Wu, Z.; Liu, Y.; Jiao, T.; Qin, Z., Simultaneously enhancing the mechanical strength and ionic conductivity of stretchable ionogels enabled by polymerization-induced phase separation. *Macromolecules* **2022,** *55* (24), 10950-10959, DOI: 10.1021/acs.macromol.2c01838

[6] Li, S.; Cheng, Y.; Zhu, H.; Xu, M.; Lv, H.; Wang, Z.; Liu, G.; Song, H., Strain-induced phase separation and mechanomodulation of ionic conduction in anisotropic nanocomposite ionogels. *ACS Appl. Mater. Interfaces* **2024,** *16* (10), 13103-13113, DOI: 10.1021/acsami.3c19167

[7] Xu, L.; Huang, Z.; Deng, Z.; Du, Z.; Sun, T. L.; Guo, Z.-H.; Yue, K., A transparent, highly stretchable, solvent-resistant, recyclable multifunctional ionogel with underwater self-healing and adhesion for reliable strain sensors. *Adv. Mater.* **2021,** *33* (51), 2105306, DOI: 10.1002/adma.202105306

[8] Qi, J.; Liu, Z.; Zhao, Z.; Tan, Z.; Liu, F.; Liu, N., A tough and reusable ionogel adhesive for flexible strain sensor. *ACS Appl. Polym. Mater.* **2024,** *6* (13), 7659-7668, DOI: 10.1021/acsapm.4c01121

[9] Yao, M.; Wu, B.; Feng, X.; Sun, S.; Wu, P., A highly robust ionotronic fiber with unprecedented mechanomodulation of ionic conduction. *Adv. Mater.* **2021,** *33* (42), 2103755, DOI: 10.1002/adma.202103755

[10] Wang, J.; Zheng, Y.; Cui, T.; Huang, T.; Liu, H.; Zhu, J.; Song, L.; Hu, Y., Bioinspired ultra-robust ionogels constructed with soft-rigid confinement space for multimodal monitoring electronics. *Adv. Funct. Mater.* **2024,** *34* (6), 2312383, DOI: 10.1002/adfm.202312383

[11] Kim, M. S.; Kim, J. H.; Yoo, H.-y.; Yoon, D.-S.; Park, D. H.; Lee, C. Y.; Kim, S. J.; Choi, S.-B.; Hong, K.; Lee, K. H., Ultrastretchable, tough, and highly conductive ionogels for multipurpose motion monitoring. *ACS Mater. Lett.* **2024,** *6* (10), 4658-4666, DOI: 10.1021/acsmaterialslett.4c01425

[12] Ren, Y.; Guo, J.; Liu, Z.; Sun, Z.; Wu, Y.; Liu, L.; Yan, F., Ionic liquid–based click-ionogels. *Sci. Adv.* **2019,** *5* (8), eaax0648, DOI: 10.1126/sciadv.aax0648

[13] Li, T.; Wang, Y.; Li, S.; Liu, X.; Sun, J., Mechanically robust, elastic, and healable ionogels for highly sensitive ultra-durable ionic skins. *Adv. Mater.* **2020,** *32* (32), 2002706, DOI: 10.1002/adma.202002706

[14] Cho, K. G.; An, S.; Cho, D. H.; Kim, J. H.; Nam, J.; Kim, M.; Lee, K. H., Block copolymer-based supramolecular ionogels for accurate on-skin motion monitoring. *Adv. Funct. Mater.* **2021,** *31* (36), 2102386, DOI: 10.1002/adfm.202102386

[15] Tan, H.; Sun, L.; Huang, H.; Zhang, L.; Neisiany, R. E.; Ma, X.; You, Z., Continuous melt spinning of adaptable covalently cross-linked self-healing ionogel fibers for multi-functional ionotronics. *Adv.*

Mater. **2024,** *36* (13), 2310020, DOI: 10.1002/adma.202310020
[16] Xie, J.; Li, X.; Liu, J.; Su, F.; Gao, R.; Zhang, C.; Liang, J.; Ji, G.; Yao, D.; Zheng, Y., A transparent and robust ionogel prepared via phase separation for sensitive strain sensing. *J. Mater. Chem. A* **2024,** *12* (26), 16160-16173, DOI: 10.1039/D4TA02305F
[17] Cheng, Y.; Zhu, H.; Li, S.; Xu, M.; Li, T.; Yang, X.; Song, H., Stretchable, low-hysteresis, and recyclable ionogel by ionic liquid catalyst and mixed ionic liquid-induced phase separation. *ACS Sustainable Chem. Eng.* **2023,** *11* (41), 15031-15042, DOI: 10.1021/acssuschemeng.3c03791
[18] Tang, W.; Dong, K.; Chen, Z.; Duan, Y.; Sun, Q.; Li, X.; Zhai, D.; Lv, T.; Chen, T., A microphase-separation ionogel electrolyte for highly stretchable all-solid-state supercapacitors. *Chem. Eng. J.* **2024,** *501*, 157726, DOI: 10.1016/j.cej.2024.157726
[19] Xu, S.; Wu, S.; Jia, J.; Li, W.; Gong, C.; Hu, X.; Qiu, Z.; Zhu, R.; Yan, Y., A transparent and robust ionogel with stress-induced microphase separation property and crack insensitivity for visual force sensor. *Small* **2025,** *21* (4), 2407026, DOI: 10.1002/smll.202407026
[20] Chen, J.; Wang, Y.; Li, L.; Miao, Y.-E.; Zhao, X.; Yan, X.-P.; Zhang, C.; Feng, W.; Liu, T., Visible-light transparent, ultrastretchable, and self-healable semicrystalline fluorinated ionogels for underwater strain sensing. *ACS Appl. Mater. Interfaces* **2023,** *15* (12), 16109-16117, DOI: 10.1021/acsami.3c02243
[21] Liu, J.; Jiang, B.; Ji, J.; Cheng, F.; Cai, C.; Fu, Y., Unmatched resilience and fatigue resistance in a novel cellulose-derived ionic gel with hierarchical superstructured synergy for advanced human-computer interaction. *Chem. Eng. J.* **2024,** *497*, 154672, DOI: 10.1016/j.cej.2024.154672
[22] Yu, N.; Liu, P.; Lin, Y.; Zhang, A., The robustness waterproof ionogel based on the phase separation to form soft hard heterostructures and the interaction of cation-π realizes underwater adhesion and sensing. *Chem. Eng. J.* **2024,** *497*, 155046, DOI: 10.1016/j.cej.2024.155046
[23] Li, X.; Song, X.; Qie, X.; Feng, H.; Min, Z.; Zhang, J.; Ren, S.; Ren, J., Tough and highly stretchable multifunctional ionogels based on phase-separated structure and nanocellulose macromolecular covalent cross-linker. *Cell Rep. Phys. Sci.* **2024,** *5* (7), 102073, DOI: 10.1016/j.xcrp.2024.102073
[24] Zhao, W.; Zheng, Y.; Huang, A.; Jiang, M.; Wang, L.; Zhang, Q.; Jiang, W., Metal-halogen interactions inducing phase separation for self-healing and tough ionogels with tunable thermoelectric performance. *Adv. Mater.* **2024,** *36* (30), 2402386, DOI: 10.1002/adma.202402386
[25] Hsiao, C.-H.; Chen, F.-F.; Zhang, C.-Z.; Lin, K.-C.; Yu, S.-S., 3d printing of hydrophobic eutectogels toughened by polymer-solvent hydrogen bonding and in situ phase separation for capacitive sensors. *Adv. Mater. Technol.* **2025,** *10* (11), 2401325, DOI: 10.1002/admt.202401325
[26] Yu, Z.; Ma, B.; Wang, J., Ion induced ultra-tough single-network ionogel. *Chem. Commun.* **2024,** *60* (91), 13388-13391, DOI: 10.1039/D4CC04732J
[27] Hong, B.; Xu, Y.; Tan, J.; Xie, Z.; Zheng, S. Y.; Wang, Q.; Zhou, Z.; Yang, J., Skin-mimetic tough polyurethane ionogel for use in soft ionotronics. *J. Mater. Chem. C* **2023,** *11* (46), 16168-16176, DOI: 10.1039/D3TC03661H
[28] Wei, C.; Bai, G.; Yu, S.; Ding, X.; Yang, W.; Zhu, S.; Wang, S.; Lu, H., Ultra-stretchable, toughened composite ionogel via synergetic hydration and hydrogen bonding interaction. *Compos. Commun.* **2024,** *48*, 101900, DOI: 10.1016/j.coco.2024.101900
[29] Qu, M.; Lv, Y.; Ge, J.; Zhang, B.; Wu, Y.; Shen, L.; Liu, Q.; Yan, M.; He, J., Hydrophobic and multifunctional strain, pressure and temperature sensor based on tpu/sio2-ils ionogel for human motion monitoring, liquid drop monitoring, underwater applications. *Colloids Surf., A* **2023,** *664*, 131103, DOI: 10.1016/j.colsurfa.2023.131103
[30] Zhao, H.; Xiao, X.; Xing, H.; Jia, X.; Jin, S., Synthesis of an ultrathin, self-adhesive, tough, and

frigostable bp@pvp/tpu ionogel for strain sensors by electrospinning. *Mater. Today Chem.* **2024,** *38*, 102102, DOI: 10.1016/j.mtchem.2024.102102

[31] Zhou, X.; Chen, J.; Zhang, W.; Zhao, W.; Wang, Q.; Peng, X., Flexible sensor based on self-assembly of halloysite nanotubes in ionic liquids/thermoplastic polyurethane composites. *Polym. Test.* **2025,** *150*, 108931, DOI: 10.1016/j.polymertesting.2025.108931

[32] Qu, M.; Zhu, M.; Lv, Y.; Liu, Q.; Li, J.; Gao, Y.; Sun, C.-L.; He, J., Hydrophobic tpu/cnts-ils ionogel as a reliable multimode and flexible wearable sensor for motion monitoring, information transfer, and underwater sensing. *ACS Appl. Mater. Interfaces* **2024,** *16* (27), 35626-35638, DOI: 10.1021/acsami.4c08196

[33] Zhao, H.; Xiao, X.; Zhou, L.; Jin, S.; Jia, X., Multifunctional flexible fibrous ionogel with superior breathability, stretchability, antimicrobial and self-adhesive properties for wearable electronics. *ACS Appl. Polym. Mater.* **2025,** *7* (13), 8684-8698, DOI: 10.1021/acsapm.5c01371

[34] Zhao, H.; Xiao, X.; Jin, S.; Jia, X., Breathable, durable, and ultrathin ionogel fiber sensors for strain and temperature sensing based on tpu and few-layer black phosphorus. *Mater. Today Nano* **2025,** *29*, 100599, DOI: 10.1016/j.mtnano.2025.100599
